# MgCuZnAl-Layered Double Hydroxides for Cr(VI) Adsorption: Effect of Interlayer Anions on Uptake Behavior and pH-Dependent Stability

**DOI:** 10.3390/ma19163366

**Published:** 2026-08-07

**Authors:** Agnieszka Lipke, Agnieszka Gładysz-Płaska, Gabriele Klydziute, Grzegorz Wójcik, Renata Łyszczek, Halina Głuchowska, Ewa Skwarek, Denis Sokol, Marek Majdan, Aivaras Kareiva

**Affiliations:** 1 Institute of Chemistry, Faculty of Chemistry and Geosciences, Vilnius University, Naugarduko St.24, 03225 Vilnius, Lithuania; gabriele.klydziute@chgf.stud.vu.lt (G.K.); denis.sokol@chgf.vu.lt (D.S.); aivaras.kareiva@chgf.vu.lt (A.K.); 2 Institute of Chemical Sciences, Faculty of Chemistry, Maria Curie-Sklodowska University in Lublin, Maria Curie-Skłodowska Sq. 2, 20-031 Lublin, Poland; agnieszka.gladysz-plaska@mail.umcs.pl (A.G.-P.); grzegorz.wojcik2@mail.umcs.pl (G.W.); renata.lyszczek@mail.umcs.pl (R.Ł.); halina.gluchowska@mail.umcs.pl (H.G.); ewa.skwarek@mail.umcs.pl (E.S.); majdan.marek8@gmail.com (M.M.)

**Keywords:** tetracationic LDH, interlayer anions, chromate(VI), adsorption mechanism, interfacial interactions, XPS, MgCuZnAl

## Abstract

**Highlights:**

- Tetracationic Mg–Cu–Zn–Al LDH developed for Cr(VI) adsorption.
- Interlayer anions control adsorption capacity and structural stability.
- Coupled adsorption–leaching analysis reveals pH-dependent durability.
- Cr(VI) uptake involves anion exchange and surface complexation.

**Abstract:**

Layered double hydroxides (LDHs) are functional materials with tunable structural, surface, and adsorption properties. In this study, multicationic LDHs were synthesised and evaluated as adsorbents for chromate(VI). Among the investigated materials, the tetracationic Mg_2_Cu_0.5_Zn_0.5_Al_1_ composition exhibited consistently high mass-normalised Cr(VI) uptake in the screening experiments and was therefore selected for detailed investigation. Therefore, its chloride and carbonate forms were selected as model materials to examine the role of interlayer anions in Cr(VI) uptake and pH-dependent stability. The chloride form exhibited a higher adsorption capacity (70 mg/g) than the carbonate form (34 mg/g), indicating that more weakly bound interlayer anions may increase the accessibility of adsorption sites and promote anion exchange during adsorption. In contrast, the carbonate form showed greater structural stability, as confirmed by reduced cation leaching over a wide pH range. The behaviour of Cr(VI) in equilibrium solution was analysed as a function of its initial concentration, which allowed a simple description of Cr(VI) sorption over a wide range of initial pH values. XRD, FTIR, and XPS analyses supported partial interlayer anion exchange and changes in the coordination environment of surface metal centres after Cr(VI) sorption. Adsorption energies of 13–16.4 kJ/mol further suggested that Cr(VI) uptake cannot be assigned to a single interaction type but involves electrostatic interactions, anion exchange, and surface complexation. These results demonstrate that the interlayer anion is a key factor governing both the Cr(VI) adsorption efficiency and aqueous stability of LDH-based adsorbents.

## 1. Introduction

Hexavalent chromium (Cr(VI)) is a highly toxic and mobile contaminant commonly found in industrial effluents. It originates mainly from processes such as electroplating, metallurgy, chemical processing, and the combustion of fossil fuels, and it poses a well-documented threat to human health and the environment [1,2,3,4,5,6,7,8]. In aqueous systems, depending on pH, Cr(VI) occurs mainly in the form of CrO_4_^2−^, HCrO_4_^−^, and Cr_2_O_7_^2−^, which makes its removal difficult due to their stability and high solubility [9,10,11]. Consequently, the effective removal of Cr(VI) from water remains a significant challenge. Layered double hydroxides (LDHs) are widely studied as adsorbents for anions due to their positively charged brucite layers and the presence of exchangeable interlayer anions. Their general structure, [M^2+^_1−*x*_M^3+^*_x_*(OH)_2_]*^x^*^+^(A*^m^*^−^)_*x*/*m*_∙*z*H_2_O, provides compositional flexibility and the ability to regulate surface properties [12,13,14,15,16]. Consequently, LDHs can remove chromates in various ways, including through electrostatic interactions, interlayer anion exchange, and surface complexation [10,17]. Additional studies have also addressed surface interactions and the dispersion of LDHs in various systems [18,19,20].

Despite extensive research into LDH-based systems for Cr(VI) adsorption, most studies have focused primarily on adsorption capacity, while the relationship between interlayer anion chemistry, the adsorption mechanism, and structural stability remains poorly understood [10]. In particular, the role of interlayer anions in controlling both interfacial reactivity and material stability under varying pH conditions remains insufficiently described. Furthermore, polycationic LDHs containing transition metals such as Cu^2+^ and Zn^2+^ have attracted attention due to their ability to modify surface reactivity and adsorption properties. These cations influence the layer formation, interlayer spacing, and redox activity of LDHs [13,21,22]. However, systematic studies combining multicationic composition with controlled interlayer chemistry remain limited, especially in the context of adsorption mechanisms rather than only performance indicators.

The Cr(VI) content varies depending on the type of effluent, the industrial process, and the sampling point. Cr(VI) concentrations in effluents from electroplating and leather tanning typically range from 3 to 30 mg/L [23]. By contrast, significantly higher concentrations may be found in process streams. For example, in actual effluent collected from electroplating plants, Cr(VI) concentrations of 280.9 mg/L and 1000 mg/L were determined [24,25]. In contaminated groundwater, maximum Cr(VI) concentrations of 2.6, 5.8, and 0.33 mg/L, respectively, were recorded for the Hinkley, Topock, and El Mirage contamination plumes [26]. By way of comparison, the WHO guideline for total chromium in drinking water is 0.05 mg/L, whilst Directive (EU) 2020/2184 sets the current parametric value at 0.05 mg/L, which is to be reduced to 0.025 mg/L by 12 January 2036 [27,28]. The regulatory values given refer to total chromium and not specifically to Cr(VI).

The initial concentration of 0.5 mmol/L used in this study corresponds to approximately 26 mg Cr/L. It therefore falls within the range of concentrations reported for certain types of industrial effluent containing Cr(VI), but it is significantly higher than the concentrations typically found in drinking water and most contaminated groundwater. The selected concentration should therefore be regarded as a model system, applicable primarily to the treatment of industrial effluent rather than to the treatment of drinking water. It was chosen to ensure measurable equilibrium concentrations and to enable a quantitative comparison of the adsorption capacities of the LDHs under investigation.

In this work, multication LDHs were synthesised and evaluated as chromate(VI) adsorbents. Based on its consistently high mass-normalised Cr(VI) uptake under the screening conditions, the tetracationic Mg–Cu–Zn–Al material was selected for detailed analysis. The study focuses on how the type of interlayer anion influences the adsorption behaviour and structural stability of this LDH during Cr(VI) uptake. Particular attention was paid to comparing interfacial interactions in chloride and carbonate forms, which reflect the differences in interlayer binding strength and exchangeability. To investigate the relationship between sorption mechanisms (including anion exchange and surface complexation) and material stability, adsorption, structural (XRD, BET), and surface (XPS) analyses were combined with leaching experiments. This approach allows for a more comprehensive understanding of LDH behaviour by linking interfacial processes with structural stability, rather than focusing solely on adsorption capacity.

## 2. Materials and Methods

### 2.1. Reagents

Mg(NO_3_)_2_ · 6H_2_O (CarlRoth, Karlsruhe, Germany), Al(NO_3_)_3_ · 9H_2_O (CarlRoth), Cu(NO_3_)_2_ · 2.5H_2_O (SigmaAldrich, St. Louis, MO, USA), Fe(NO_3_)_3_ · 9H_2_O (SigmaAldrich), Zn(NO_3_)_2_ · 6H_2_O (CarlRoth), Na_2_CO_3_ (CarlRoth), NaOH (CarlRoth), NaCl (POCh, Gliwice, Poland), HCl 36% (POCh), H_2_SO_4_ 95% (POCh), K_2_CrO_4_ (SigmaAldrich), Na_2_HPO_4_ (POCh), 1,5-Diphenylcarbazide (SigmaAldrich). All chemical reagents used were of analytical grade without additional purification.

### 2.2. Synthesis of LDHs

**Figure 1:**
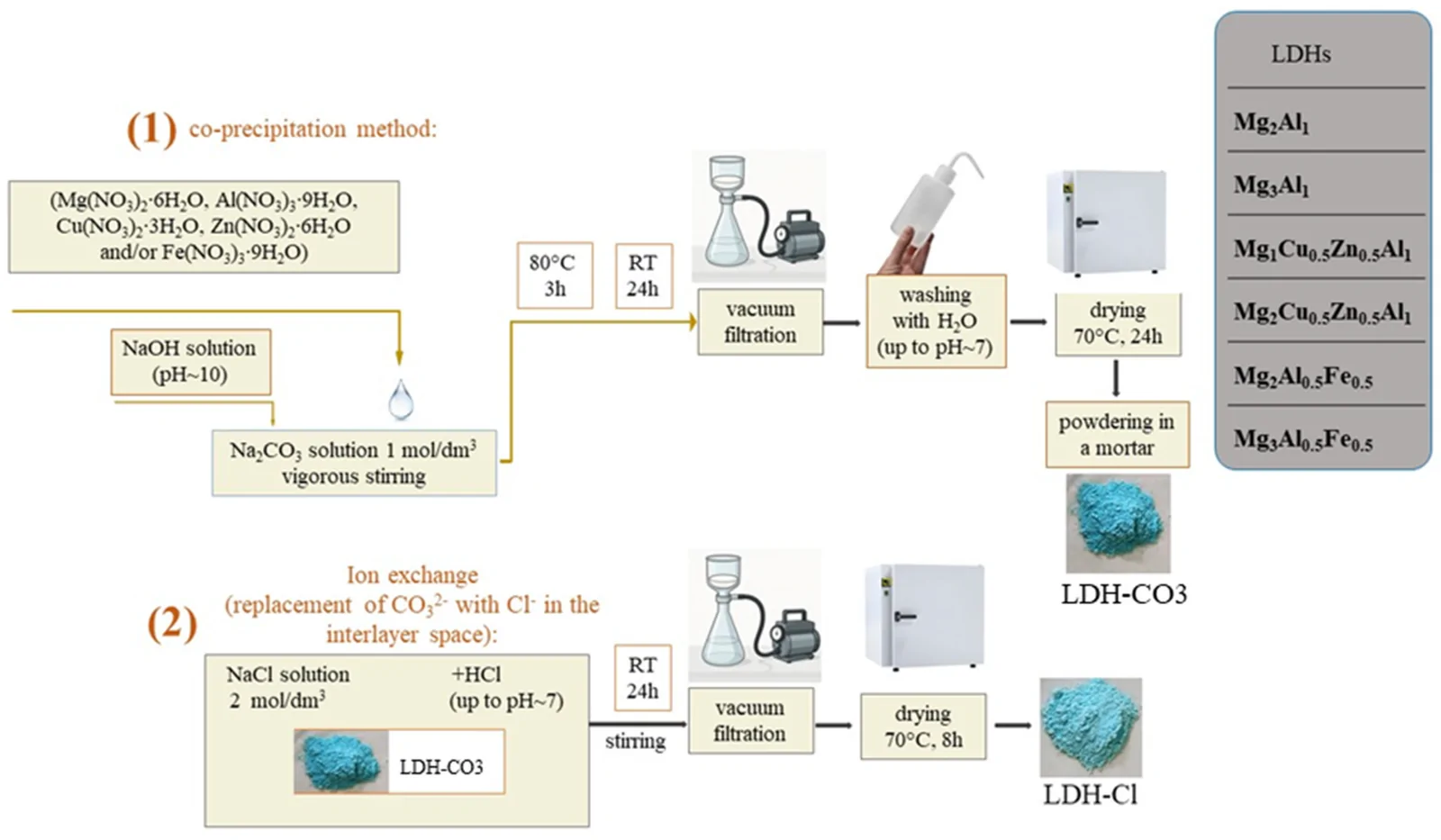
Schematic diagram showing the main steps of LDH synthesis by the co-precipitation method, leading to the formation of chloride- and carbonate-intercalated LDHs. (Photographs of powdered LDH-Cl and LDH-CO3 are shown for Mg_2_Cu_0.5_Zn_0.5_Al_1_-Cl and Mg_2_Cu_0.5_Zn_0.5_Al_1_-CO_3_ as representative examples).

LDH materials (Mg_2_Al_1_, Mg_3_Al_1_, Mg_1_Cu_0.5_Zn_0.5_Al_1,_ Mg_2_Cu_0.5_Zn_0.5_Al_1_, Mg_2_Al_0.5_Fe_0.5_, Mg_3_Al_0.5_Fe_0.5_) were synthesised by the co-precipitation method. The cationic solution (150 mL), composed of appropriate amounts of metal nitrates (Mg(NO_3_)_2_∙6H_2_O, Al(NO_3_)_3_∙9H_2_O, Cu(NO_3_)_2_∙3H_2_O, Zn(NO_3_)_2_∙6H_2_O, and/or Fe(NO_3_)_3_∙9H_2_O), was prepared and added dropwise to Na_2_CO_3_ solution (1 mol/L, 100 mL) under stirring (250 rpm). The solution was maintained at a pH of about 10 using the NaOH solution. Next, the mixture was stirred, initially while heating at 80 °C for 3 h and then at room temperature for 24 h. The solution was vacuum filtered, and the precipitate was washed with distilled water (to neutral pH). The precipitate was dried in an oven at 70 °C for 24 h and then ground in a mortar (Figure 1). In this way, all layered double hydroxides in carbonate form (LDH-CO3) were obtained.

Anion exchange was performed in 250 mL of a 2  mol/L NaCl solution with addition of diluted hydrochloric acid (to maintain pH at 7). One gram of LDH-CO3 was used. The reaction was carried out at room temperature with stirring for 24 h. The final products, layered double hydroxides in chloride form (LDH-Cl), were obtained by vacuum filtration, followed by drying for 10 h at 70 °C.

### 2.3. Sorption Experiments

Sorption of chromate(VI) ions on the obtained LDH materials was carried out in a system containing 0.025 g of the sorbent sample and 25 mL of K_2_CrO_4_ solution with a concentration of 0.5 mmol/L and pH = 7.2. An initial pH of 7.2 was chosen as a neutral reference condition, which limits the dissolution of LDH in an acidic environment and avoids the strong competition from OH^−^ ions observed under alkaline conditions. An initial Cr(VI) concentration of 0.5 mmol/L was chosen as a model concentration, which ensured measurable equilibrium concentrations, allowed differentiation between the LDHs under investigation, and provided a sufficient amount of chromium for sorption characterisation. The sorption process took place at room temperature. The shaking time was 1, 2, or 24 h for preliminary (screening) tests and 1 h for the studies on isotherms and the effect of pH. A mechanical shaker was used to contact the phases. After the specified time, the systems were filtered. The concentration of Cr(VI) in the initial and equilibrium solutions, as well as in the eluates obtained after desorption, was determined using a selective spectrophotometric method involving 1,5-diphenylcarbazide. After acidifying the samples and adding 1,5-diphenylcarbazide, the absorbance of the resulting coloured complex was measured at a wavelength of 543 nm using a JASCO V-660 UV–Vis spectrophotometer (JASCO Corporation, Hachioji, Tokyo, Japan), in a cuvette with a path length of 1 cm, against a blank sample. The Cr(VI) concentration was determined on the basis of an external calibration curve prepared from K_2_CrO_4_ standard solutions in the range of 0.05–1.00 mg Cr/L. Samples whose concentration exceeded the calibration range were diluted appropriately prior to measurement. For selected representative filtrates, the total concentration of dissolved chromium was additionally determined by ICP-OES as a check on the spectrophotometric results. The ICP-OES determinations referred to total chromium and did not allow for the distinction between the different oxidation states of chromium. In the samples analysed by both methods, the total chromium concentrations determined by ICP-OES were, within the limits of experimental uncertainty, comparable to the Cr(VI) concentrations determined by the 1,5-diphenylcarbazide method. This indicates that there was no measurable proportion of dissolved forms of chromium with an oxidation state other than Cr(VI). Calculations concerning the sorption of chromate ions were performed based on the following equations:

(1) $$c_{s}=\frac{c_{in}-c_{eq}}{m}\cdot V,$$

(2) $$\%A=\frac{c_{in}-c_{eq}}{c_{in}}\cdot 100\%,$$

where: *c_s_* refers to the amount of adsorbed Cr(VI) (mol/g), *c_in_* is the concentration of Cr(VI) ions in the initial solution, *c_eq_* is the concentration of Cr(VI) ions in the solutions after sorption (mol/L), %*A* is the percentage of sorption, *m* is the mass of the corresponding LDH expressed in g, and *V* is the volume of the solution used for sorption (L). All sorption experiments were performed in triplicate, and the values shown in the figures represent the mean of three independent sorption experiments. The experimental deviation did not exceed ±3%.

### 2.4. Characterization Techniques

The synthesised adsorbents were characterized by thermal analyses applying the thermogravimetric (TG) and differential scanning calorimetry (DSC) methods using the SETSYS16/18 analyzer (Setaram Instrumentation, KEP Technologies Group, Caluire, France). The samples (about 8–10 mg) were heated in the alumina open crucibles in the temperature range of 30–1000 °C at a heating rate of 10 °C/min in the dynamic air atmosphere (v = 0.75 L/h).

Fourier transform infrared spectroscopy (FTIR) of obtained materials was performed using an Alpha spectrometer (Bruker, Inc., Ettlingen, Germany) in the wavenumber range from 4000 to 450 cm^−1^, with a resolution of 4 cm^−1^.

Powder X-ray diffraction (XRD) analysis (step width of 0.01°, scan speed 5°/min, 8–80° 2θ angle range) on Rigaku MiniFlex II diffractometer (Rigaku Corporation, Tokyo, Japan) using Cu-Kα radiation (λ = 1.5419 Å) was used to study the crystallinity and phase purity of synthesised LDH samples.

The specific surface area was measured by the Brunauer–Emmet–Teller (BET) method under vacuum for degassed at 120 °C samples by N_2_ adsorption–desorption isotherm (at 77 K) using a TriStar II instrument (Micromeritics Instrument Corporation, Norcross, GA, USA). The pore size distribution of the material produced was obtained using the Barrett–Joyner–Halenda (BJH) method.

X-ray photoelectron spectroscopy (XPS) studies were cunducted using the multi-chamber UHV system (PREVAC, Rogów, Poland). Spectra were collected using hemispherical Scienta R4000 electron analyser (Scienta Omicron, Uppsala, Sweden). Scienta SAX-100 X-ray source (Al Kα, 1486.6 eV, 0.8 eV band) equipped with the XM 650 X-Ray Monochromator (0.2 eV band) were used as a complementary equipment. The pass energy of the analyser was set to 200 eV for survey spectra (with 750 meV step), and 50 eV for regions (high resolution spectra): C1s, O1s, Zn2p, Al2p, Cr2p, and Cu2p (with 100 meV step). The base pressure in the analysis chamber was 5 · 10^−9^ mbar. During the spectra collection, it was not higher than 1 · 10^−8^ mbar. The oxidation state of chromium bound to the solid phase after adsorption was assessed on the basis of the binding energies and spectral components identified in the high-resolution Cr 2p XPS spectra.

Zeta potential was measured by electrophoresis using the Zetasizer Nano ZS90 apparatus by Malvern Instruments Ltd. (Malvern, UK). The zeta potential was calculated on the basis of electrophoretic mobility using the Smoluchowski equation. The measurements were performed at 100 ppm solid concentration ultrasonication of the suspension.

The concentration of Mg, Al, Cu, and Zn ions in the solutions was determined by ICP-OES. A Varian 720-ES spectrometer (Varian, Inc., Palo Alto, CA, USA) was used for the determination. Calibration solutions of Al, Cu, Mg, and Zn ions were prepared from a multi-element Certipur^®^ ICP standard solution with a concentration of 10 mg/L. Ultrapure water was obtained using a Polwater DL-2 demineralization system (Labopol, Kraków, Poland). Quantitative elemental analysis of the solid MgCuZnAl-LDH samples was performed using a Perkin Elmer Optima 7000 DV inductively coupled plasma optical emission spectrometer (ICP-OES, Perkin-Elmer, Waltham, MA, USA) (Table 1). A blank sample was used—5% HNO_3_ nitric acid solution. Standard solutions of all analyzed metals (Mg, Al, Cu, Zn) were also used (single-element ICP standard, 1000 mg/L, Roth).

All analyses described above were carried out before and after the Cr(VI) ion sorption processes.

### 2.5. Desorption Study

Mg_2_Cu_0.5_Zn_0.5_Al_1_ samples in carbonate and chloride forms, saturated with chromate(VI) ions, were used for desorption experiments. In each experiment, 0.025 g of the sorbent were contacted with 25 mL of the eluent solution. Aqueous solutions of Na_2_CO_3_, NaCl, NaOH, and Na_2_HPO_4_ at a concentration of 0.01 mol/L, as well as distilled water, were applied as eluents. The desorption process was carried out at room temperature under continuous shaking for 1 h. Subsequently, the suspensions were filtered, and the concentration of Cr(VI) released into the eluates was determined using the selective 1,5-diphenylcarbazide spectrophotometric method described in Section 2.3. Therefore, the reported desorption values refer specifically to desorbed Cr(VI), rather than to total chromium. The pH of the eluents was measured before (pH_in_) and after (pH_eq_) the desorption process.

Percentage desorption (%D) of chromate(VI) ions was calculated using the following equation:

(3) $$\%D=\frac{n_{aq}}{n_{s}}\cdot 100\%$$

where *n_aq_* is the number of moles of Cr(VI) released into the solution during a given desorption step, and *n_s_* is the number of moles of Cr(VI) present in the sorbent sample before that desorption step.

The reusability of the studied sorbents was evaluated in three consecutive adsorption–desorption cycles. In each cycle, adsorption was carried out from a 0.5 mmol/L Cr(VI) solution (pH = 7.2) for 1 h. Subsequently, desorption was performed using a 10 mmol/L NaOH solution, with a solid–liquid contact time of 1 h. The Cr(VI) concentration in the filtrates collected after each adsorption and desorption step was determined as described in Section 2.3.

## 3. Results and Discussion

### 3.1. Adsorption Behaviour and Isotherm Analysis

**Figure 2:**
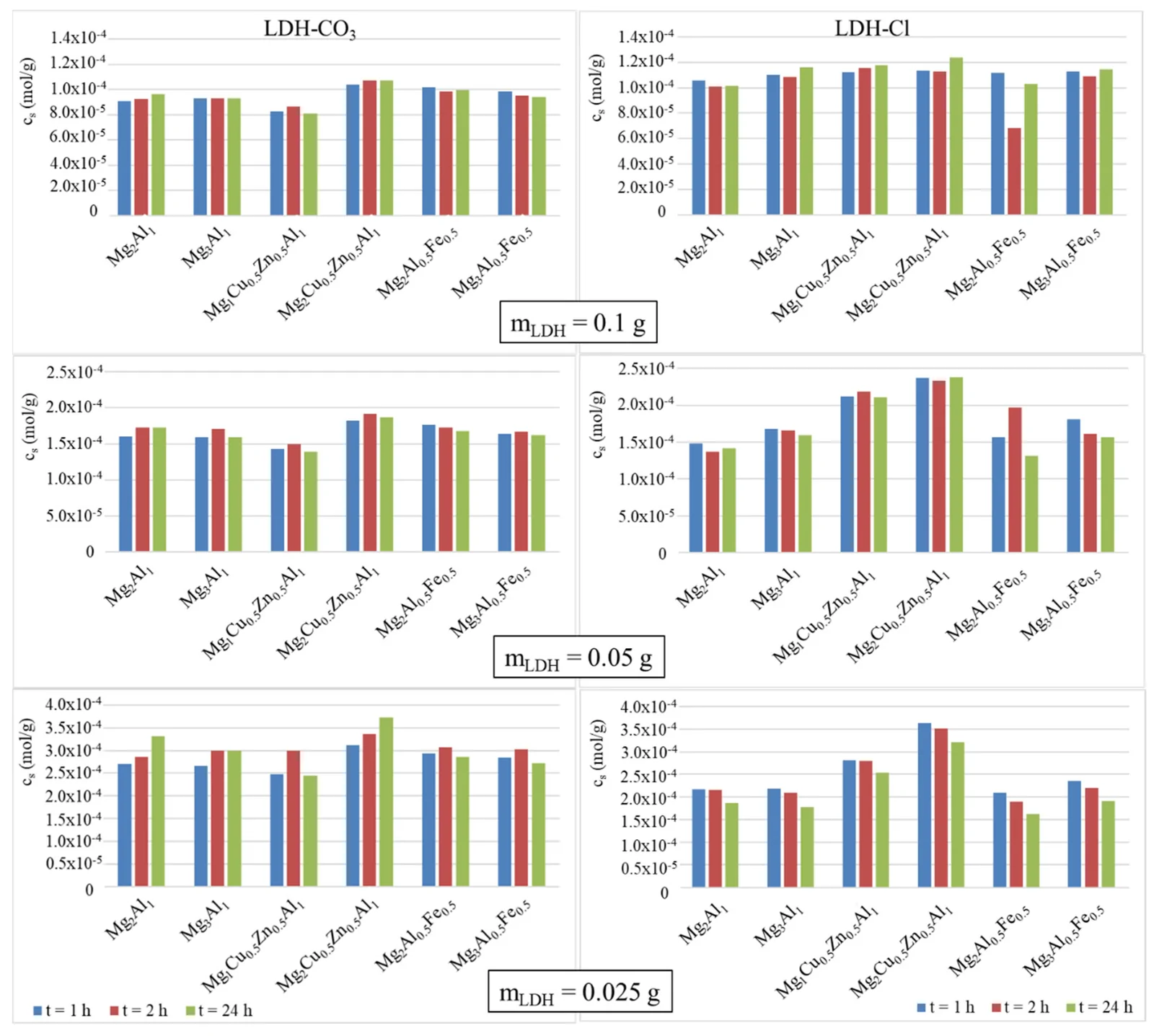
Comparison of the efficiency of the Cr(VI) anion sorption process on all synthesized LDH materials, both in chloride and carbonate form; *c_in_* = 0.5 mmol/L, *V* = 25 mL, pH = 7.2, t = 1, 2, 24 h, and the mass of the adsorbent used (*m* = 0.025, 0.05, 0.1 g).

Six layered double hydroxides (Mg_2_Al_1_, Mg_3_Al_1_, Mg_1_Cu_0.5_Zn_0.5_Al_1_, Mg_2_Cu_0.5_Zn_0.5_Al_1_, Mg_2_Al_0.5_Fe_0.5_, Mg_3_Al_0.5_Fe_0.5_) were obtained by co-precipitation. This method was chosen for synthesis in order to avoid the calcination process. Due to the presence of copper in the LDH structure, the calcination process could lead to the formation of stable oxide phases (in the range of 400–500 °C) [29]. In the presence of zinc and aluminium, the resulting copper oxide CuO can form spinel phases, which causes an irreversible loss of the LDH memory effect. The LDH obtained by us was used for the sorption of Cr(VI) ions. Initially, screening tests were carried out using all six LDHs in order to select the most effective sorbent. The form of the adsorbent (LDH-CO3 or LDH-Cl), the weight of the sample (0.025, 0.05 or 0.1 g), and the phase contact time (1 or 2 or 24 h) were taken into account (Figure 2). The adsorption processes of chromate(VI) anions were successfully carried out on all synthesised materials. In general, the chloride forms exhibited a higher Cr(VI) uptake by mass than their corresponding carbonate forms. LDHs containing Cu and Zn consistently exhibited high adsorption values, particularly at the lowest sorbent dose, for which the differences between the compositions under investigation were most pronounced. The criterion for selecting the material for further investigation was the amount of adsorbed Cr(VI), expressed as *c_s_* [mol/g]. The differences between the LDHs under investigation were most pronounced at the lowest sorbent mass of 0.025 g. Under these conditions, the *c_s_* values obtained for Mg_2_Cu_0.5_Zn_0.5_Al_1_–CO_3_ were 3.1 · 10^−4^, 3.3 · 10^−4^, and 3.7 · 10^−4^ mol/g after 1, 2, and 24 h. For Mg_2_Cu_0.5_Zn_0.5_Al_1_–Cl, the corresponding values were 3.6 · 10^−4^, 3.5 · 10^−4^, and 3.2 · 10^−4^ mol/g. Thus, this composition exhibited consistently high Cr(VI) adsorption in both interlayer forms and over all contact times studied. Based on the screening experiments, Mg_2_Cu_0.5_Zn_0.5_Al_1_ was selected as a representative, highly efficient, multi-cation LDH for further studies on Cr(VI) adsorption and the influence of the interlayer anion.

Based on the ICP-OES analysis and after normalization to Al = 1, the actual cationic layer composition of the precipitated LDH-CO3 form of this sample was determined as [Mg_1.88_Cu_0.51_Zn_0.50_Al_1_(OH)_7.78_]^+^, showing very good agreement with the theoretical structure [Mg_2_Cu_0.5_Zn_0.5_Al_1_(OH)_8_]^+^. In turn, for the anion-exchanged sample, the composition was determined as [Mg_1.79_Cu_0.47_Zn_0.49_Al_1_(OH)_7.50_]^+^. The material retained the characteristic LDH structure, with only slight changes in cation composition suggesting limited layer destabilization. For the sake of simplicity, throughout the remainder of the article, this sorbent will be denoted as Mg2CuZnAl-CO3 and Mg2CuZnAl-Cl for the carbonate and chloride forms, respectively.

**Figure 3:**
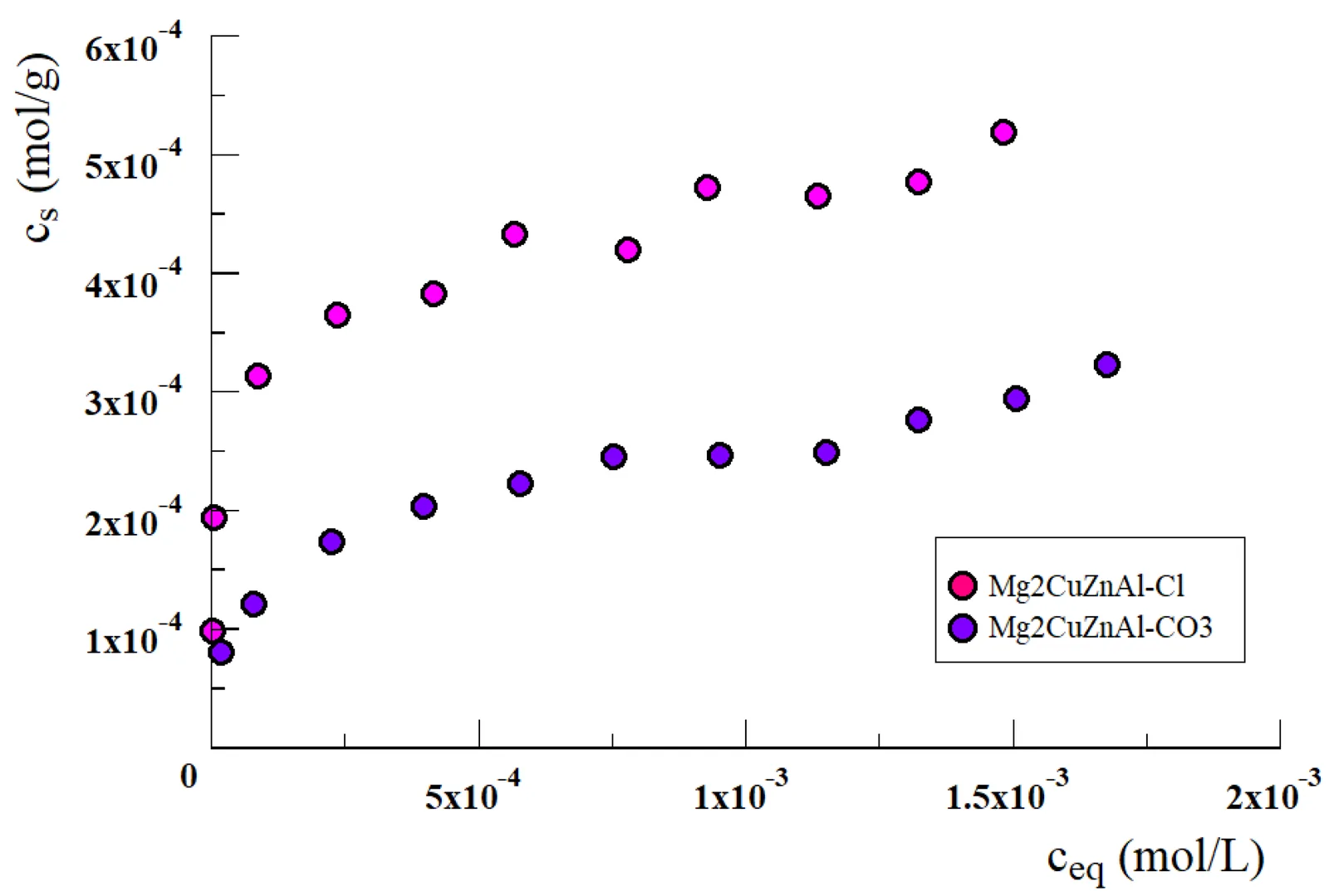
Changes in the sorption quantity on Mg2CuZnAl-Cl and Mg2CuZnAl-CO3 forms depending on the equilibrium concentration of Cr(VI).

Detailed sorption studies have shown that Mg2CuZnAl-Cl is a better chromium sorbent, with the sorption capacity value being 1.35 mmol/g, compared to the Mg2CuZnAl-CO3 form, which is 0.65 mmol/g (see Figure 3 and Figure S1—Supplementary Information). Adsorption isotherms were evaluated based on the following models: Redlich-Peterson, Langmuir-Freundlich, Freundlich, Langmuir, Temkin, and Dubinin Radushkevich [24]. The results presented in Table 2 indicate that the sorption process is complex.

The Redlich-Peterson equation is used as a compromise between the Langmuir and Freundlich equations. This equation contains three parameters and is presented as follows:

c_s_ = A · c_eq_/(1 + B · c_eq_^g^), (4)

where *A* (L/g) and *B* (L/mol) denote isothermal constants, while *g* refers to the exponent characterising the heterogeneity of the sorbent surface. The lower the value of *g*, the more heterogeneous the surface. Systems in which chloride ions are replaced by chromate ions as a result of ion exchange, i.e., Mg2CuZnAl-Cl + Cr(VI), are characterised by lower surface heterogeneity compared to Mg2CuZnAl-CO3 + Cr(VI) systems. The isotherm of the Mg2CuZnAl-Cl + Cr(VI) system is more “steep”, i.e., there is a sudden increase in *c_s_* values with an increase in *c_eq_*. The value of the exponent *g* is very close to unity. In general, *c_s_* values for chloride systems are higher than *c_s_* values for carbonate systems, which results from the easy replacement of monovalent Cl^−^ ions by Cr(VI) ions. At the same time, the values of the *g* parameter are higher for the chloride system compared to the carbonate one, which indicates greater surface heterogeneity in the case of the carbonate system.

The Langmuir-Freundlich model describes sorption on heterogeneous surfaces. At low adsorbate concentrations, this model simplifies to the Freundlich model, while at high adsorbate concentrations, it simplifies to the Langmuir model. It is described by the equation:

c_s_ = a_L-F_ (K_L-F_ · c_eq_)^n^/(1 + K_L-F_ · c_eq_)^n^, (5)

where *a*_*L*-*F*_ denotes the sorption capacity of the sorbent in mol/g, and *K*_*L*-*F*_ is the adsorption constant expressed in L/mol. The exponent *n* refers to surface heterogeneity. The values of the *n* parameter are higher for the chloride system compared to the carbonate one, so similarly to the Redlich-Peterson model [30], we can conclude about the greater heterogeneity of the sorbent surface in the carbonate form.

The Freundlich isotherm describes sorption processes occurring on heterogeneous surfaces. This isotherm provides an expression that defines surface heterogeneity and the exponential distribution of active sites on the surface and their energy:

c_s_ = K_F_ · c_eq_^1/n^, (6)

where *K_F_* denotes the sorption capacity of the sorbent, and 1/*n* refers to surface heterogeneity. If the value of *n* is between 0 and 10, this indicates a favourable adsorption process [31].

The Langmuir isotherm model is widely used in sorption studies, especially when describing the sorption of metal ions on solid surfaces. The main assumption of the model is monolayer sorption on the surface without interactions between sorbed complexes. It is assumed that the surface is homogeneous with sorption sites of equal energy. This model is described by the equation:

c_s_ = a_L_ · K_L_ · c_eq_/(1 + K_L_ · c_eq_), (7)

where *a_L_* is the capacity of the sorption monolayer expressed in mol/L, and *K_L_* is the sorption constant expressed in L/mol. In our case, the Langmuir model describes the sorption behaviour of chromates very poorly on both the chloride and carbonate forms due to the energetic differences of the sorption sites.

The Temkin isotherm model considers the effect of indirect interactions between the adsorbate and the adsorbent on the heat of adsorption. This model is based on the assumption that the heat of adsorption decreases linearly with surface coverage and that adsorption is characterised by a homogeneous distribution of binding energy up to the maximum binding energy. This contrasts with the Freundlich model, where the decrease in the heat of adsorption is logarithmic [32,33].

Temkin’s model is based on the equation:

c_s_ = B ∙ lnc_eq_ + B ∙ lnK_T_, (8)

where:

B = R · T/b_T_, (9)

with *R* referring to the gas constant 8.31 J/mol · K, *T* denoting the absolute temperature, *b_T_* referring to the heat of adsorption expressed in J/mol, and *K_T_* relating to the adsorption constant expressed in L/mg.

In the tested systems, the adsorption heat *b_T_* takes on low values, which is related to the ion-exchange nature of the adsorption process [34]. At the same time, it is easy to notice that the adsorption heat is lower for the system with Mg2CuZnAl-Cl compared to Mg2CuZnAl-CO3, which is related to the charge of the adsorbing and desorbing ions.

The Dubinin-Radushkevich isotherm is a mathematical model used to describe adsorption and is very useful for characterising the mechanism of metal ion adsorption. It allows the distribution of energy on heterogeneous sorbent surfaces to be assessed. This model allows physical adsorption to be distinguished from chemical adsorption based on the value of the average free energy of adsorption [35].

The Dubinin-Radushkevich model is based on the equation:

ln(c_s_) = ln(Q_m_) − K_D-R_ · ε^2^, (10)

where *Q_m_* denotes the adsorption capacity of the sorbent, *K*_*D*-*R*_ is a constant allowing the determination of the average free energy of adsorption expressed in mol^2^/J^2^, and *ε* is the adsorption potential, described by the equation:

ε = R · T · ln(1 + 1/c_eq_). (11)

Based on the *K*_*D*-*R*_ value, the average free energy of adsorption *E* can be calculated:

E = 1/(2 K_D-R_)^0.5^. (12)

The adsorption energy values calculated from the Dubinin–Radushkevich equation and presented in Table 2 (E = 13–16.4 kJ/mol) indicate that the sorption of Cr(VI) on Mg2CuZnAl layered double hydroxides proceeds via a complex mechanism [36]. It largely involves physical interactions, such as electrostatic and van der Waals forces, especially in the case of chromate ion adsorption on Mg2CuZnAl-CO3. It also involves chemisorption, including the formation of Cr–O–M coordination bonds, as well as partial anion exchange in the LDH interlayer. The latter case applies in particular to the sorption of chromates on Mg2CuZnAl-Cl. These observations indicate that both the chemical nature and the type of interlayer anion determine the complex mechanism of Cr(VI) adsorption on the tested materials.

Additionally, the difference in maximum chromium adsorption capacity between Mg2CuZnAl-Cl (70 mg/g) and Mg2CuZnAl-CO3 (34 mg/g) calculated based on the Langmuir-Freundlich isotherm equation indicates that the type of interlayer anion significantly affects the nature of the Cr(VI) sorption process. At the same time, it confirms the complex mechanism determined on the basis of Dubinin-Radushkevich adsorption energy. In the case of chromate ion adsorption on Mg2CuZnAl-CO3, we observe a lower maximum capacity (34 mg/g) due to the limited exchangeability of strongly bound carbonate anions. Meanwhile, the sorption process associated with ion exchange reactions, which particularly applies to Mg2CuZnAl-Cl, leads to a higher sorption capacity (70 mg/g).

### 3.2. Effect of pH

**Figure 4:**
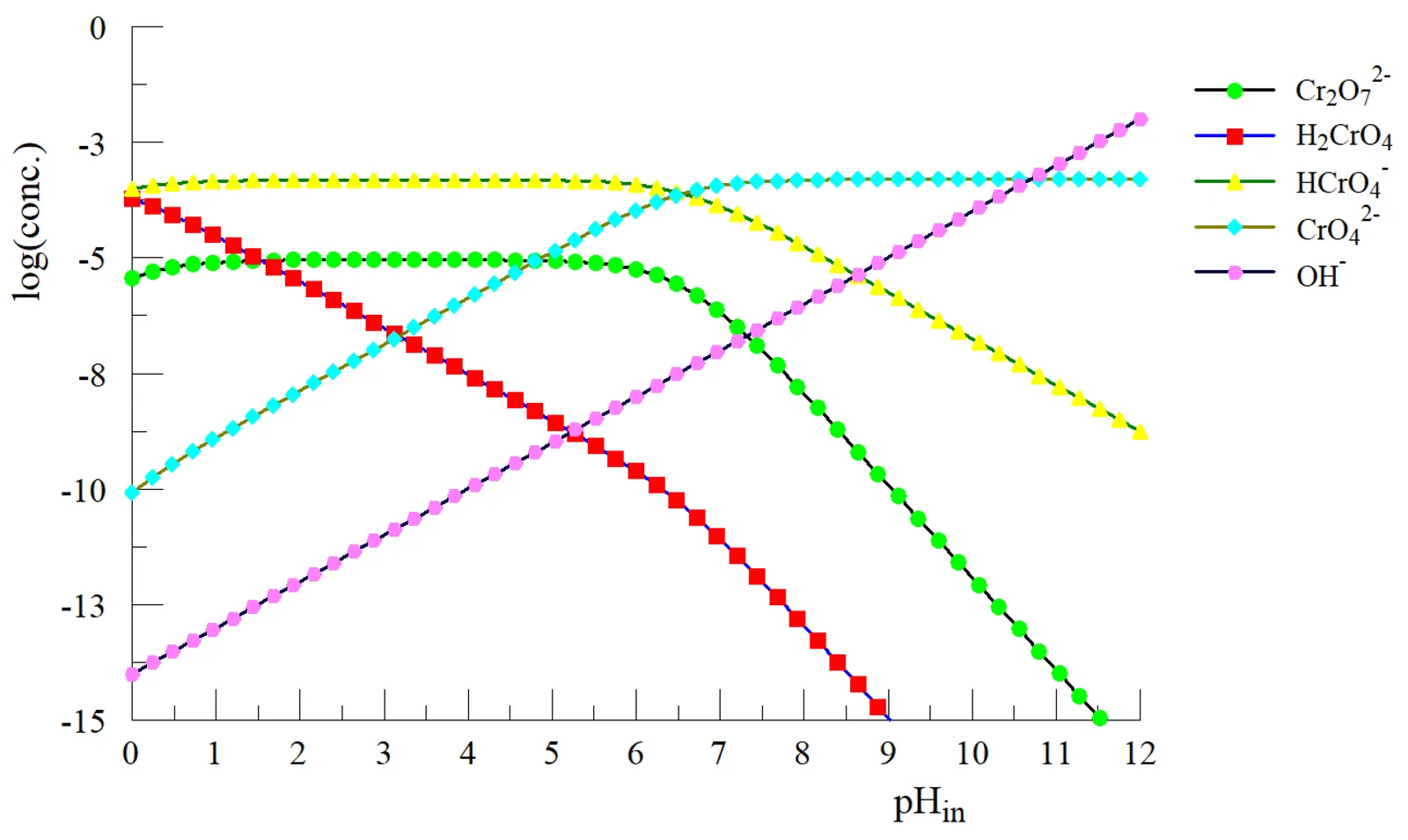
Speciation of chromium(VI) ions in aqueous solutions depending on pH ([Cr(VI)_t_] = 0.5 mmol/L).

**Figure 5:**
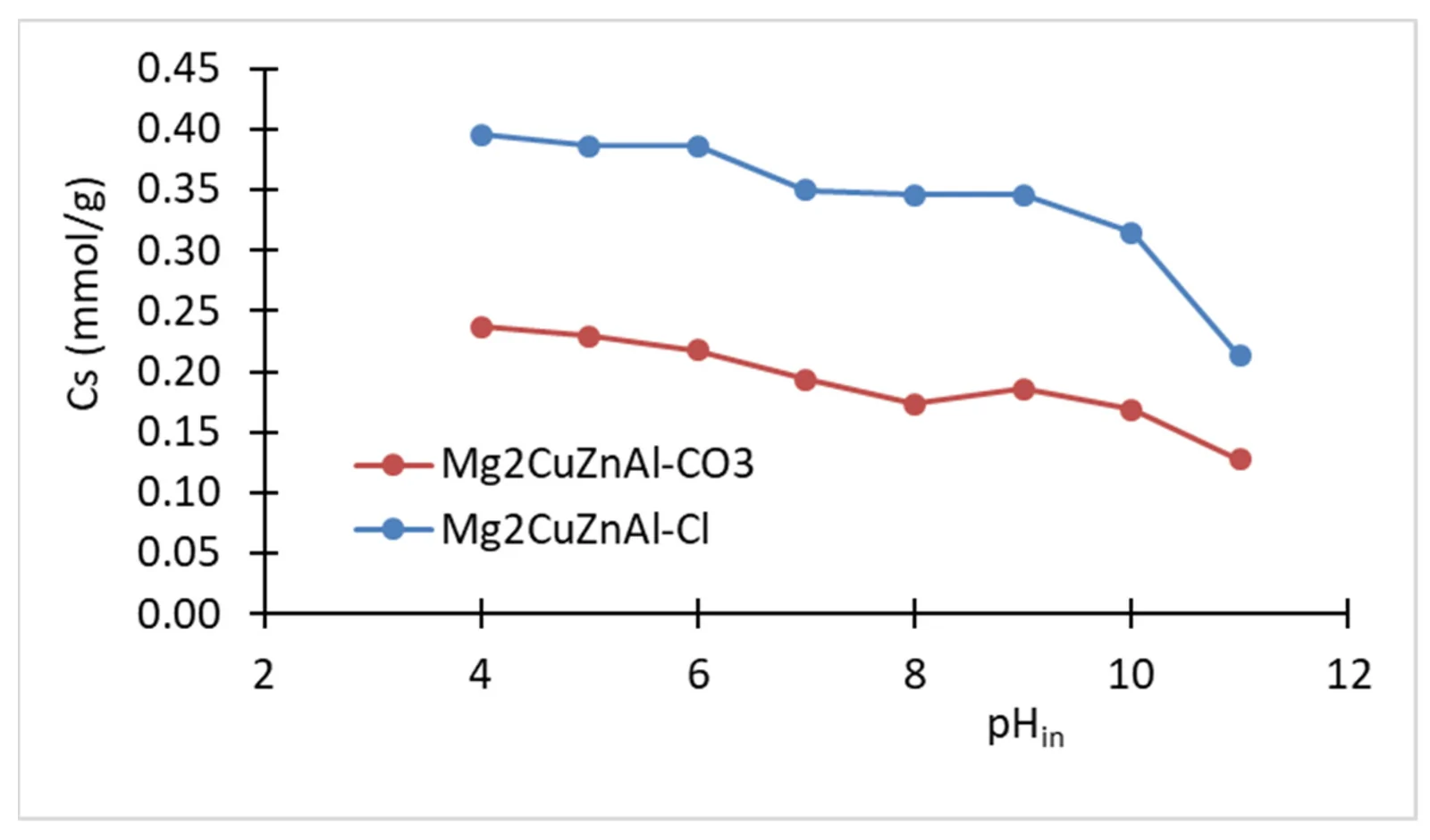
Changes in the sorption quantity on Mg2CuZnAl-Cl and Mg2CuZnAl-CO3 forms depending on the pH of the initial Cr(VI) solution.

Solution pH is a key factor controlling Cr(VI) adsorption, as it affects both the aqueous speciation of chromium(VI) (Figure 4) and the surface charge of LDHs. The sorption of Cr(VI) onto chloride and carbonate forms of Mg2CuZnAl was investigated over an initial pH range of 4–11. The highest sorption quantities were observed under acidic conditions, while adsorption decreased with increasing pH (Figure 5). This behaviour can be attributed to the combined effects of Cr(VI) speciation, progressive deprotonation of the LDH surface, increasing competition from OH^−^ ions for adsorption sites under alkaline conditions, and possible structural rearrangements of the LDH [37,38].

Adsorption proceeds primarily through ion exchange and surface interactions. The exchange with hydroxide ions can be described by:

Mg2CuZnAl-Cl + OH^−^ → Mg2CuZnAl-OH + Cl^−^ (13)

Mg2CuZnAl-CO3 + 2OH^−^ → Mg2CuZnAl-(OH)_2_ + CO_3_^2−^ (14)

In the carbonate form, the strongly bound CO_3_^2−^ anions limit exchange processes due to their high charge and strong interaction with the hydroxide layers. As a result, chromate exchange is restricted:

Mg2CuZnAl-CO3 + 2HCrO_4_^−^ → Mg2CuZnAl-(HCrO_4_)_2_ + CO_3_^2−^ (15)

In contrast, the chloride form exhibits higher exchangeability due to weaker binding of Cl^−^:

Mg2CuZnAl-Cl + HCrO_4_^−^ → Mg2CuZnAl-HCrO_4_ + Cl^−^ (16)

In addition to ion exchange, chromate ions can form surface complexes with metal centres (Mg^2+^, Zn^2+^, Cu^2+^, Al^3+^) indicating a mixed adsorption mechanism involving both electrostatic interactions and chemisorption [39,40].

### 3.3. Surface Charge and Zeta Potential

**Figure 6:**
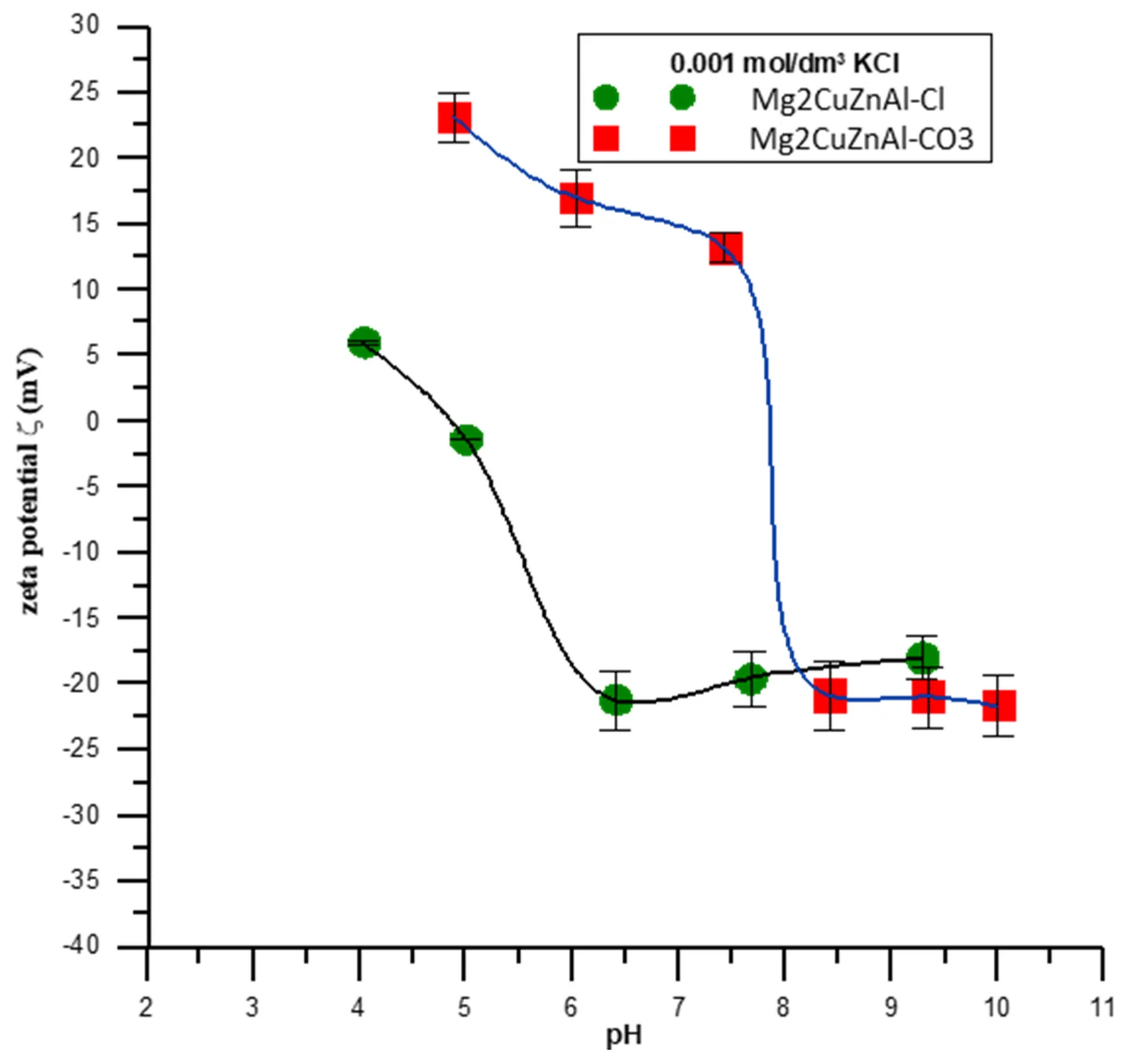
Zeta potential (ζ) vs. pH for Mg2CuZnAl–Cl and Mg2CuZnAl–CO3 measured in 0.001 mol/L KCl.

**Figure 7:**
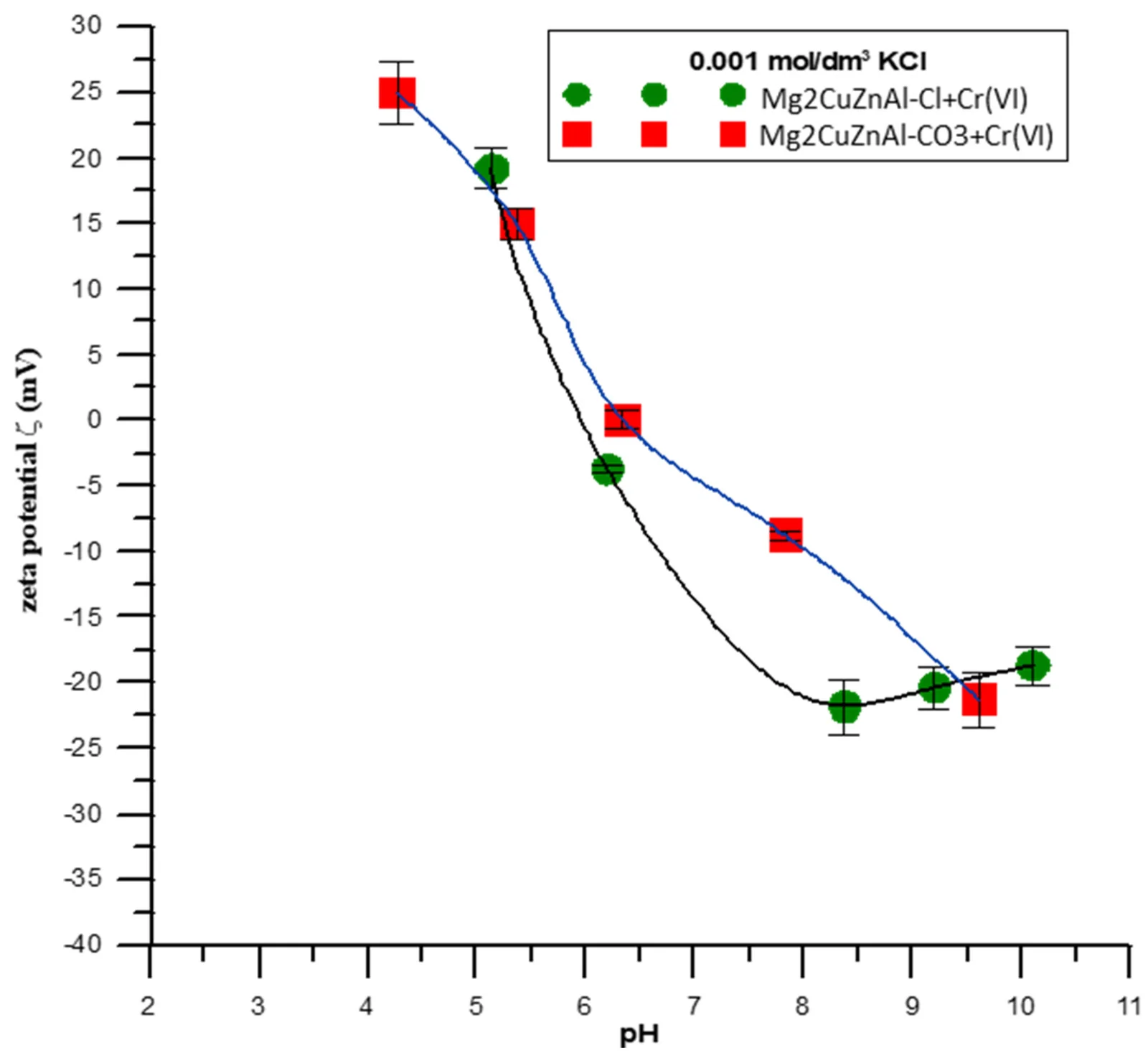
Zeta potential (ζ) vs. pH for Mg2CuZnAl–Cl and Mg2CuZnAl–CO3 after Cr(VI) sorption process measured in 0.001 mol/L KCl.

The zeta potential (ζ) of Mg2CuZnAl–Cl and Mg2CuZnAl–CO3 was measured as a function of pH (Figure 6 and Figure 7). Both materials show a decrease in ζ with increasing pH, reflecting progressive surface deprotonation of hydroxyl groups.

The chloride form exhibits lower ζ values and a lower isoelectric point (pH_IEP_ ≈ 5.3), indicating weaker binding of interlayer anions and higher surface reactivity. In contrast, the carbonate form shows higher positive ζ at low pH and a significantly higher isoelectric point (pH_IEP_ ≈ 7.6), consistent with stronger binding of CO_3_^2−^ and greater surface stability. This behaviour reflects differences in the structure and compactness of the electrical double layer.

After Cr(VI) adsorption, both materials exhibit a shift towards negative ζ values (−20 to −25 mV at pH ≥ 8), indicating strong modification of the surface by chromate species. The isoelectric point shifts towards lower pH, reflecting surface complexation and partial displacement or masking of interlayer anions.

The convergence of ζ values for both materials at higher pH suggests that Cr(VI) adsorption dominates surface charge behaviour regardless of the initial interlayer anion. This leads to the formation of a stable, negatively charged surface, which directly influences adsorption efficiency, aggregation behaviour, and interactions with other ions in the solution.

### 3.4. Effect of pH on Elemental Release in LDH–Solution Systems

**Figure 8:**
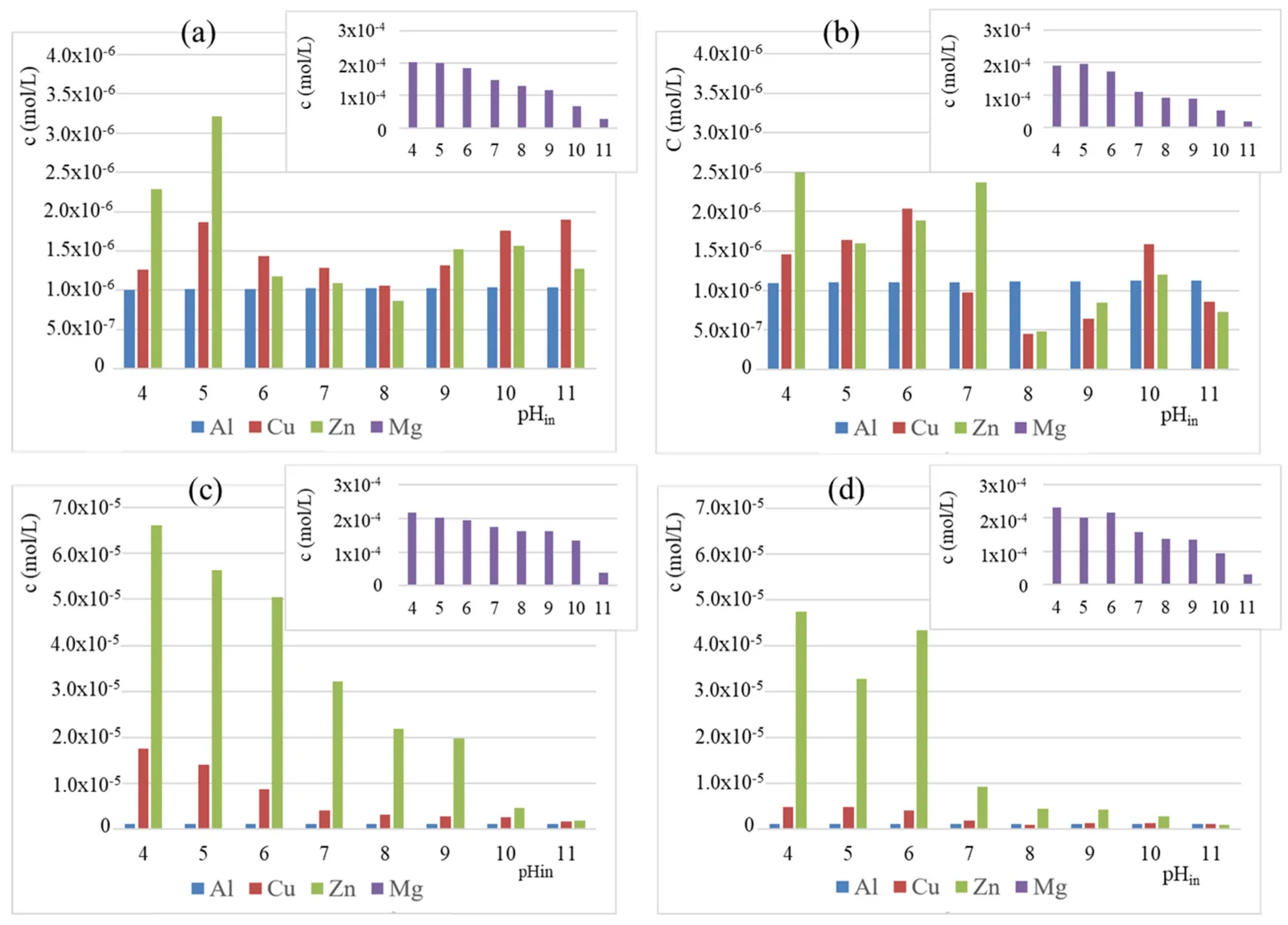
Stability of the LDH structure depending on pH: Mg2CuZnAl-CO3 (**a**), Mg2CuZnAl-CO3 + Cr(VI) (**b**), Mg2CuZnAl-Cl (**c**), Mg2CuZnAl-Cl + Cr(VI) (**d**).

Figure 8 shows the concentration of magnesium, copper, zinc, and aluminium in solutions with different initial pH values (pH_in_) after contact with the corresponding LDH: Mg2CuZnAl-CO3 (a), Mg2CuZnAl-CO3 + Cr(VI) (b), Mg2CuZnAl-Cl (c), Mg2CuZnAl-Cl + Cr (VI) (d). Analysis shows that in all systems, c(Mg) decreases significantly with increasing pH, which indicates a decrease in LDH dissolution and, at the same time, an increase in stability of brucite layers in neutral and alkaline solutions. Mg^2+^ is the weakest bound of all metal cations in the layer. Therefore, its degree of leaching is the highest in the entire analyzed pH range. The decrease in magnesium content in solutions ranges from 2.03 · 10^−4^ to 2.88 · 10^−5^ mol/L for Mg2CuZnAl-CO3, from 1.90 · 10^−4^ to 1.79 · 10^−5^ mol/L for Mg2CuZnAl-CO3 + Cr(VI), from 2.18 · 10^−4^ to 3.75 · 10^−5^ mol/L for Mg2CuZnAl-Cl, and from 2.30 · 10^−4^ to 2.99 · 10^−5^ mol/L for Mg2CuZnAl-Cl + Cr(VI). The most stable system is therefore Mg2CuZnAl-CO3 + Cr(VI). The chloride form of LDH is clearly less stable, as can be seen not only from the magnesium concentration but significantly lower contents of zinc and copper are present in solutions contacted with the LDH-CO3 than with the LDH-Cl. The introduced chloride anions destabilize the hydrogen bond network, thereby “loosening” the LDH layers and facilitating the leaching of Cu^2+^ and Zn^2+^. At pH = 4, the zinc concentration is 30 times higher, and the copper concentration is 10 times higher in solutions for chloride LDH than for carbonate one. In an acidic environment, water molecules and H^+^ ions penetrate the network more strongly, resulting in protonation of hydroxyl groups in the octahedral layers and increased leaching of metal cations. In the case of aluminium, its concentration remained at approximately 10^−6^ mol/L and varied only slightly over the entire investigated pH range, regardless of the LDH form. In contrast to the divalent cations, this behaviour is consistent with incongruent dissolution of the LDH layers, involving preferential release of Mg^2+^, Cu^2+^, and Zn^2+^ and retention of Al in the solid phase or in an Al-enriched surface layer. The low dissolved Al concentration therefore does not itself demonstrate extensive Al dissolution followed by precipitation of a separate secondary phase. Nevertheless, the formation of a poorly crystalline or amorphous Al-bearing phase cannot be completely excluded, as no additional crystalline phase was detected by XRD. The addition of chromate (VI) ions increases the stability of both LDH forms, especially in alkaline solutions, and this is more pronounced in the case of the Mg2CuZnAl-Cl + Cr(VI) material. As a result of the exchange of Cl^−^ anions for chromate(VI) ions, the electrostatic bonds between the layers are strengthened and the matrix structure is stiffened.

### 3.5. Desorption Study

The desorption of chromate anions adsorbed on carbonate and chloride forms of Mg2CuZnAl was investigated. 0.01 M solutions of Na_2_CO_3_, NaCl, NaOH, and Na_2_HPO_4_, as well as distilled water, were used as desorbing agents. The results obtained in the form of the percentage of Cr(VI) desorption and the pH of the solutions are presented in Table 3. Analysis of these data allows us to conclude that in all systems, a significantly higher desorption from Mg2CuZnAl-Cl is observed compared to the corresponding values for the carbonate form, regardless of the pH of the solution. This confirms that the pH value is not a decisive factor in the amount of desorption, but rather the type of anion in the interlayer and the type of interaction are important. The strongest desorption capacity was demonstrated by Na_2_CO_3_, Na_2_HPO_4_, and NaOH solutions, which directly results from their greater affinity for the layered structure of double hydroxides. In the case of NaOH, desorption was 38.2% and 56.2% for the carbonate and chloride forms, respectively. This is due to the fact that strong bases can cause partial disruption of the LDH matrix, for example, by weakening the hydrogen bonds between the layers and facilitating delamination. As a result, previously bound anions are released. In addition, the pH values for the solutions before and after desorption presented in the table suggest that it is precisely the high pH that promotes desorption, possibly also through an increase in the negative charge of the LDH surface, whereby chromate anions are removed from the surface. Comparing desorption in Na_2_HPO_4_ and NaCl solutions, as well as the pH values of the solutions, it can be concluded that multivalent anions more effectively displace CrO_4_^2−^ from the interlayer space. The affinity of the anion to the LDH structure is therefore more important than the pH, which is quite similar for both solutions (pH_eq_ = 8.7 and 7.5). In turn, the very low desorption values observed in water, i.e., 4.8% obtained for the Mg2CuZnAl–Cl form and 0.2% for Mg2CuZnAl–CO3, can be attributed to the fact that although sorption and desorption occur through a simple anion-exchange mechanism (Cl^−^ ⇿ CrO_4_^2−^), the chromate anion stabilizes the LDH structure more strongly than Cl^−^ and is therefore not so easily removed. In addition, some of the CrO_4_^2−^ ions are adsorbed on the surface, as in the case of Mg2CuZnAl–CO3, and interact strongly with it, making desorption difficult [41]. The results obtained from desorption show that ion exchange and specific structural interactions have a decisive influence on the extent of desorption. These results also confirm that chromates are located in the interlayer space in the case of the Mg2CuZnAl–Cl form, while surface adsorption and stronger structural stabilization, i.e., stronger interaction with the Mg2CuZnAl–CO3 structure, is observed.

The reusability of the investigated LDH sorbents was evaluated in three consecutive adsorption–desorption cycles using NaOH as the desorbing agent (Table 4). Although Na_2_CO_3_ and Na_2_HPO_4_ showed the highest Cr(VI) desorption efficiencies in the preliminary tests, NaOH was selected to avoid introducing large amounts of phosphate anions into the interlayer and, in particular, to limit the conversion of the chloride form into a carbonate-containing form after the first regeneration cycle. Under the applied conditions, NaOH removed only part of the adsorbed Cr(VI), but both sorbents retained the ability to adsorb chromate anions in subsequent cycles. In the first cycle, Mg2CuZnAl–Cl showed substantially higher adsorption than Mg2CuZnAl–CO3, with efficiencies of 77% and 36%, respectively. The corresponding desorption efficiencies were 58% and 39%, which are consistent with the values obtained for NaOH in the preliminary desorption experiment, namely 56.2% and 38.2%. After the first regeneration, adsorption by Mg2CuZnAl–Cl decreased to 31% in the second cycle and 24% in the third cycle, whereas adsorption by Mg2CuZnAl–CO3 decreased to 25% and then remained at the same level. Desorption efficiency also gradually declined, from 58% to 29% and 13% for chloride form and from 39% to 27% and 14% for carbonate one. Thus, the initial advantage of the chloride form became less pronounced after regeneration and was no longer observed in the third cycle. The decrease in adsorption and desorption may be associated with incomplete removal of previously adsorbed Cr(VI) and the resulting reduction in the number or accessibility of available sorption sites. Overall, the results confirm the possibility of partial reuse of both Mg2CuZnAl materials, but the progressive decrease in their performance indicates that further optimization of the regeneration conditions is required before their long-term practical applicability can be assessed.

### 3.6. XRD Spectroscopy

**Figure 9:**
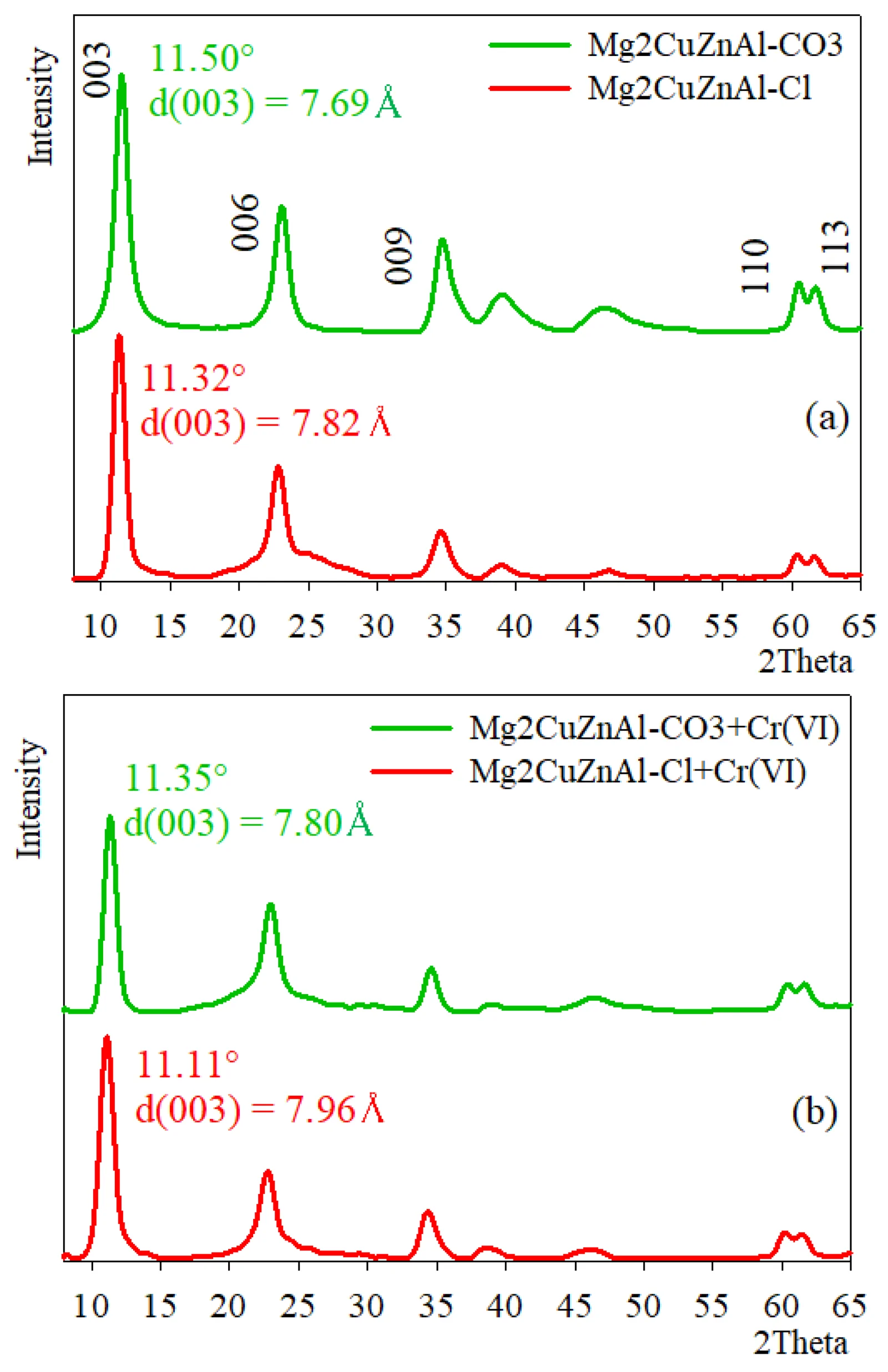
Powder XRD patterns of Mg2CuZnAl-CO3 and Mg2CuZnAl-Cl sorbents before (**a**) and after (**b**) Cr(VI) sorption process.

The XRD diffractograms of the analysed samples (Figure 9) display the characteristic LDH reflections, particularly the basal reflections (003), (006), and (009) and the high-angle reflections (110) and (113) [13,42], confirming preservation of the layered structure after conversion of the LDH-CO3 to LDH-Cl and after sorption. During Cr(VI) sorption on Mg2CuZnAl–CO3, only a slight increase in the basal spacing d(003) is observed, whereas in the Mg2CuZnAl–Cl series, this effect is more visible. Parallel shifts of the (006) and (009) reflections (Table S1—Supplementary Information) indicate that these changes mainly concern the interlayer space and are not the result of a random displacement of a single peak. According to the literature, oxyanion sorption by LDHs may involve an interlayer anion exchange mechanism, whose extent depends on the initial interlayer anion [10,41]. The limited increase in d(003) for the carbonate form is consistent with the stabilising effect of CO_3_^2−^, which restricts exchange and may favour a greater contribution of surface adsorption mechanisms. Materials containing more labile anions (e.g., Cl^−^, NO_3_^−^) are more susceptible to chromate exchange [10,40], which is consistent with the grater increase in d(003) observed for the chloride form. Slight basal spacing changes may also reflect reorganisation of the interlayer water. However, the systematic increase in d(003), especially for the chloride form, supports interlayer involvement in sorption process. In addition, the Mg2CuZnAl–Cl + Cr(VI) sample shows a clearer increase in the full width at half maximum (FWHM) of the (003) and (006) reflections, indicating a decrease in the coherence of the layer arrangement along the c-axis. This is typical of systems with mixed interlayer anions, differences in hydration, or partial interstratification [10,41]. By contrast, the (110) reflection shows only minimal shifts. The calculated lattice parameter a, corresponding to the distance between cations in the brucite layers, remains nearly constant (~3.06–3.07 Å), while parameter c increases with d(003). This indicates that the brucite-like layers remain preserved and the observed modifications mainly concern the interlayer space [13]. Furthermore, no distinct reflections of new crystalline phases (e.g., metal oxides or crystalline metal chromates) were detected. However, the absence of new XRD reflections does not exclude the formation of Cr-O-M surface complexes and does not by itself allow unambiguous distinction between intercalation and surface adsorption [10].

### 3.7. FTIR Spectroscopy

**Figure 10:**
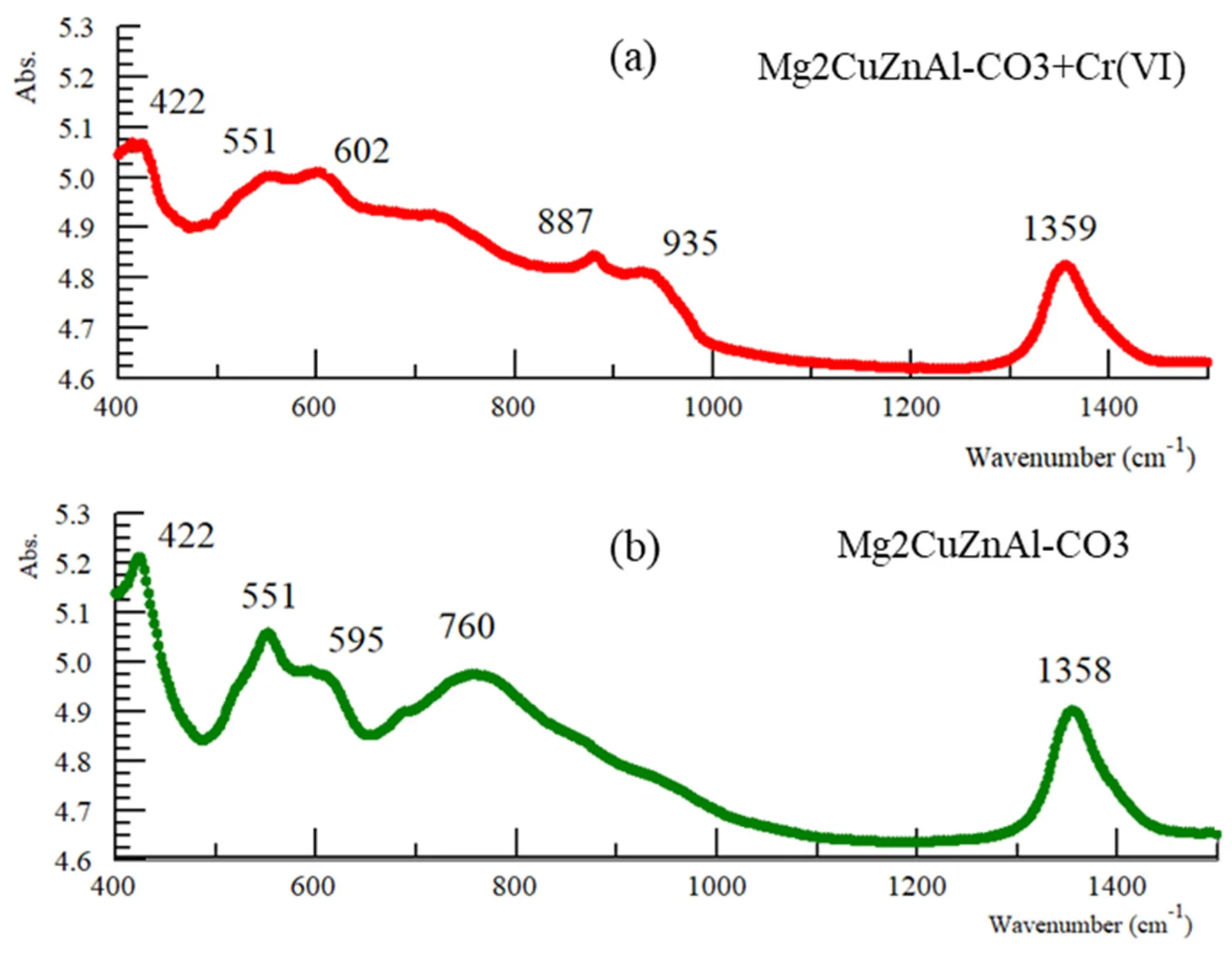
FTIR spectra of Mg2CuZnAl-CO3 with adsorbed chromate anions (**a**) and Mg2CuZnAl -CO3 (**b**).

The FTIR spectra of Mg2CuZnAl samples before and after chromate(VI) sorption shown in Figure 10 provide evidence for the presence of CrO_4_^2−^ ions in the LDH structure. In the spectrum of the Mg2CuZnAl sample saturated with chromate(VI) ions, two characteristic absorption bands appear at 887 and 935 cm^−1^. These bands can be assigned to Cr–O stretching vibrations of Cr(VI)-oxo species associated with the LDH phase. The band at around 887 cm^−1^ is consistent with the asymmetric stretching mode of chromate ions, whereas the feature at 935 cm^−1^ may indicate a distorted chromate environment, lowered symmetry of surface-associated chromate species, or the presence of hydrogen chromate-/dichromate-like species. Their positions are consistent with the literature data for chromate compounds [43,44]. The presence of these bands confirms the effective binding of chromate(VI) anions by Mg2CuZnAl-CO3. In the Mg2CuZnAl-CO3 spectrum (Figure 10b), the strong band at 1358 cm^−1^ is attributed to stretching vibrations of carbonate anions (CO_3_^2−^) from the interlayer. This band is less intense in the spectrum of the Mg2CuZnAl-CO3 + Cr(VI) sample. The lowering of the peak characteristic of the carbonate ion confirms that the CO_3_^2−^ ions have been replaced by chromate(VI) ions only to a small extent, as they are strongly bound to the LDH network. The absorption bands in the range of 400–800 cm^−1^ are caused by vibrations of the hydrotalcite structure in the LDH network, i.e., Mg-O/Al-O/O-Al-O/O-Mg-O, Al-OH, and H_2_O groups. These bands have a different arrangement for samples with Cr(VI), which indicates that the vibrations of the network are disturbed by the sorbed chromates [14,44].

### 3.8. BET Analysis

The BET analysis revealed clear differences in the porous structure of the tested LDH materials, as well as significant changes occurring after Cr(VI) sorption (Table 5). The Mg2CuZnAl-CO3 sample has the largest specific surface area (56.65 m^2^/g) and a significant pore volume (0.262 cm^3^/g), which is typical for LDHs containing carbonate ions. CO_3_^2−^ anions are quite large and divalent, so they stabilise the ordered interlayer structure, which promotes the development of porosity and high accessibility of the adsorption surface. Partial replacement of CO_3_^2−^ with smaller, monovalent Cl^−^ anions leads to a significant reduction in specific surface area—the BET value for Mg2CuZnAl-Cl is only 27.24 m^2^/g, which is almost half that of Mg2CuZnAl-CO3. Chloride ions do not provide the same stability of interlayer interactions, which leads to a more compact and compressed LDH structure. As a result, access to micropores becomes more difficult, and the aggregation of plates further reduces the total active surface area. After Cr(VI) ion sorption, both materials exhibit contrasting behaviour. In the case of Mg2CuZnAl-CO3, the specific surface area decreases from 56.65 to 47.85 m^2^/g, indicating partial pore blockage by chromate anions. At the same time, the unchanged pore volume combined with an increase in pore diameter (from 18.65 to 21.90 nm) suggests that Cr(VI) penetrates smaller pores and induces their widening or structural reorganisation without increasing their total volume. This mechanism may be related to the formation of Cr–O–M surface complexes that locally loosen or expand the layered structure, as described by Bao et al. [44]. In the case of Mg2CuZnAl-Cl, the sorption effect is reversed: the specific surface area increases from 27.24 to 30.69 m^2^/g, while the pore volume increases from 0.140 to 0.172 cm^3^/g. This indicates the opening of additional porous spaces. Chromate(VI) anions, partially penetrating the interlayer area of Mg2CuZnAl-Cl, lead to the reorganisation of aggregates, resulting in increased internal surface availability. SEM images show smaller, less compact aggregates after sorption, which is consistent with the partial deagglomeration mechanism described by Li M et al. [45]. The increase in pore diameter (from 20.62 to 22.43 nm) further confirms that Cr(VI) promotes the development of mesoporousness.

In summary, the interlayer structure and type of anions present significantly affect the porosity and sorption behaviour towards Cr(VI). Mg2CuZnAl-CO3 is characterised by a large initial surface area, but sorption mainly leads to pore blockage. In contrast, Mg2CuZnAl-Cl, despite its initially small surface area, exhibits greater structural flexibility—Cr(VI) adsorption, increased porosity, and an increase in BET surface area. Combining these data with zeta potential measurements reveals a clear relationship between pore structure and electrokinetic behaviour: Mg2CuZnAl–CO3 samples, with a larger surface area and smaller pores, exhibit higher pH_IEP_ and pH_PZC_ values compared to Mg2CuZnAl–Cl. This is attributed to the stronger binding of CO_3_^2−^ anions to the surface and a greater number of adsorption sites that stabilise the positive surface charge. In contrast, the smaller surface area and larger, less ordered pores of Mg2CuZnAl–Cl facilitate the detachment of Cl^−^ anions and faster neutralisation of the electrokinetic charge, which is reflected in lower pH_IEP_ and pH_PZC_ values. In summary, both the pore characteristics and the type of interlayer anion in LDH determine the structure of the double electric layer and the electrokinetic stability of the particles. Samples with larger surface areas and strongly bound anions (Mg2CuZnAl–CO3) form a more compact and stable double electric layer, while Mg2CuZnAl–Cl exhibits a looser and more reactive layer, sensitive to pH changes and the presence of ions in the solution.

### 3.9. Thermal Analysis

**Figure 11:**
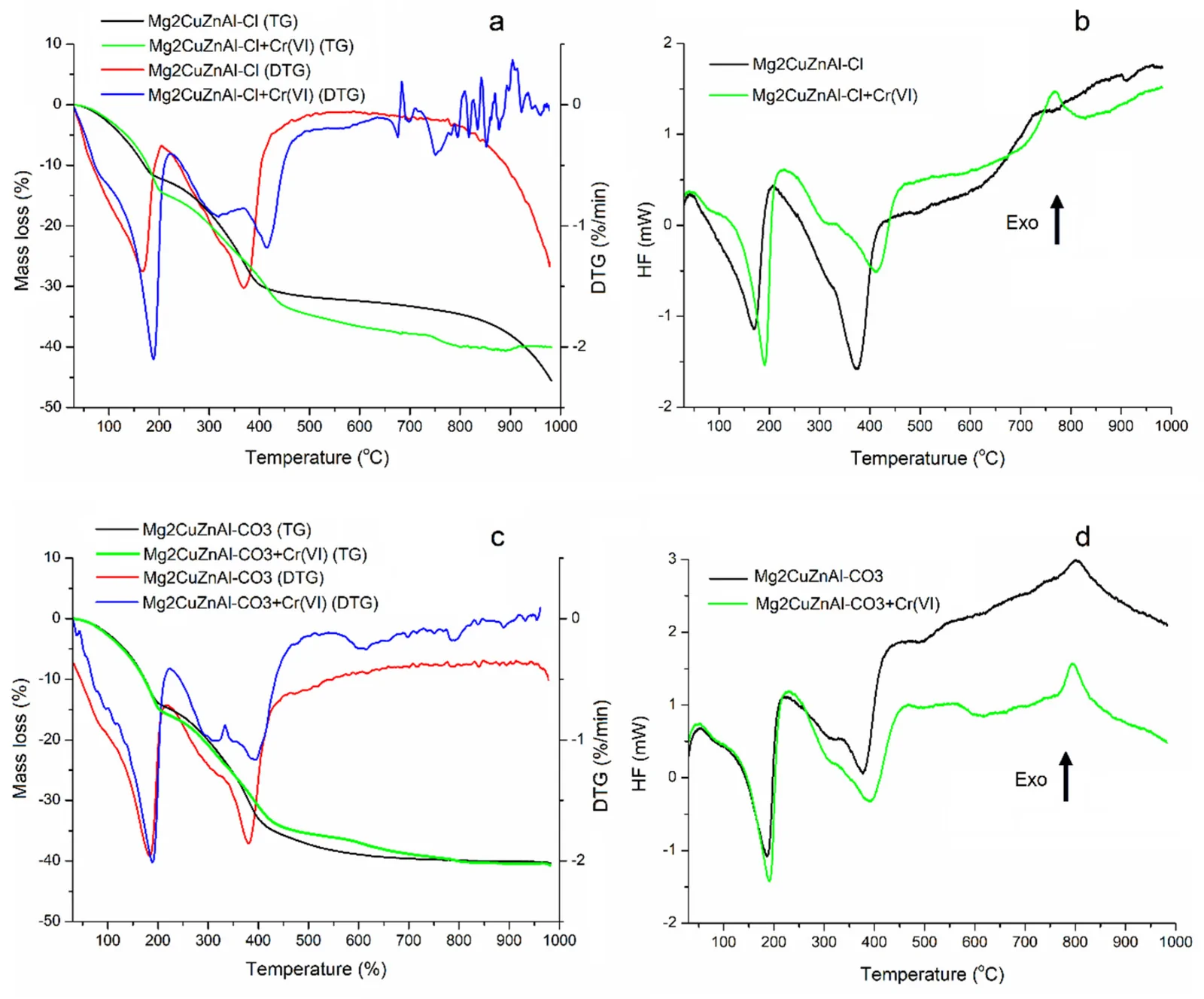
TG-DTG (**a**,**c**) and DSC (**b**,**d**) curves of Mg2CuZnAl-CO3, Mg2CuZnAl-CO3 + Cr(VI), Mg2CuZnAl-Cl, and Mg2CuZnAl-Cl + Cr(VI) recorded in air atmosphere.

The thermal behaviour of the investigated samples was examined by TG, DTG, and DSC in air atmosphere (Figure 11). The thermal decomposition of all tested samples occurs in three main stages, within the temperature ranges of about 30–200 °C, 200–450 °C, and 450–1000 °C (Figure 11a,c). The first stage is most likely related to the release of weakly bound water. The mass losses at 200 °C were 13.8, 14.7, 12.2, and 14.2% for Mg2CuZnAl-CO3, Mg2CuZnAl-CO3 + Cr(VI), Mg2CuZnAl-Cl, and Mg2CuZnAl-Cl + Cr(VI), respectively, indicating different water contents in the samples. The chromate-loaded samples show higher water content. A detailed analysis of the DTG and DSC curves below 200 °C shows that these losses result from overlapping processes, as indicated by the signal shapes. A very weak endothermic effect is visible on the DSC curves (Figure 11b,d), together with changes in the DTG profiles between 30 and about 90 °C for Mg2CuZnAl-CO3, Mg2CuZnAl-CO3 + Cr(VI), and Mg2CuZnAl-Cl + Cr(VI), which is attributed to removal of the most weakly bound water molecules located on polycrystallite surfaces. The loss of interlayer water is accompanied by a pronounced endothermic effect. For Mg2CuZnAl-Cl, the peak temperature of this endotherm is 170 °C, whereas for the other samples, it is around 190 °C. This observation suggests that partial replacement of carbonate by chloride reduces hydrogen-bond formation and weakens water binding.

The corresponding enthalpy values for Mg2CuZnAl-CO3, Mg2CuZnAl-CO3 + Cr(VI), Mg2CuZnAl-Cl, and Mg2CuZnAl-Cl + Cr(VI) are 318, 360, 266.4, and 374 J/g, respectively. The lowest value for Mg2CuZnAl-Cl is consistent with the presence of the most weakly bound water molecules. In the second stage, the mass losses 21.8, 19.8, 18.9, and 19.0% were recorded for the Mg2CuZnAl-CO3, Mg2CuZnAl-CO3 + Cr(VI), Mg2CuZnAl-Cl, and Mg2CuZnAl-Cl + Cr(VI), respectively. The DTG and DSC profiles recorded between 200 and 450 °C indicate that this stage also consists of two overlapping processes, accompanied by overlapping endothermic effects. The peak temperatures of the stronger endothermic effects were 379, 393, 374, and 412 °C, respectively. This stage is associated with water release due to layer decomposition, elimination of carbonate as CO_2_, and formation of the corresponding mixture of metal oxides. In the Mg2CuZnAl-Cl sample, some stable products, most probably metal oxychlorides, are formed and, above 900 °C, further decompose into the corresponding metal oxides. The total mass losses for Mg2CuZnAl-CO3, Mg2CuZnAl-CO3 + Cr(VI), Mg2CuZnAl-Cl, and Mg2CuZnAl-Cl + Cr(VI) are 40.0, 40.5, 45.4, and 40.1%, respectively.

### 3.10. SEM Analysis

**Figure 12:**
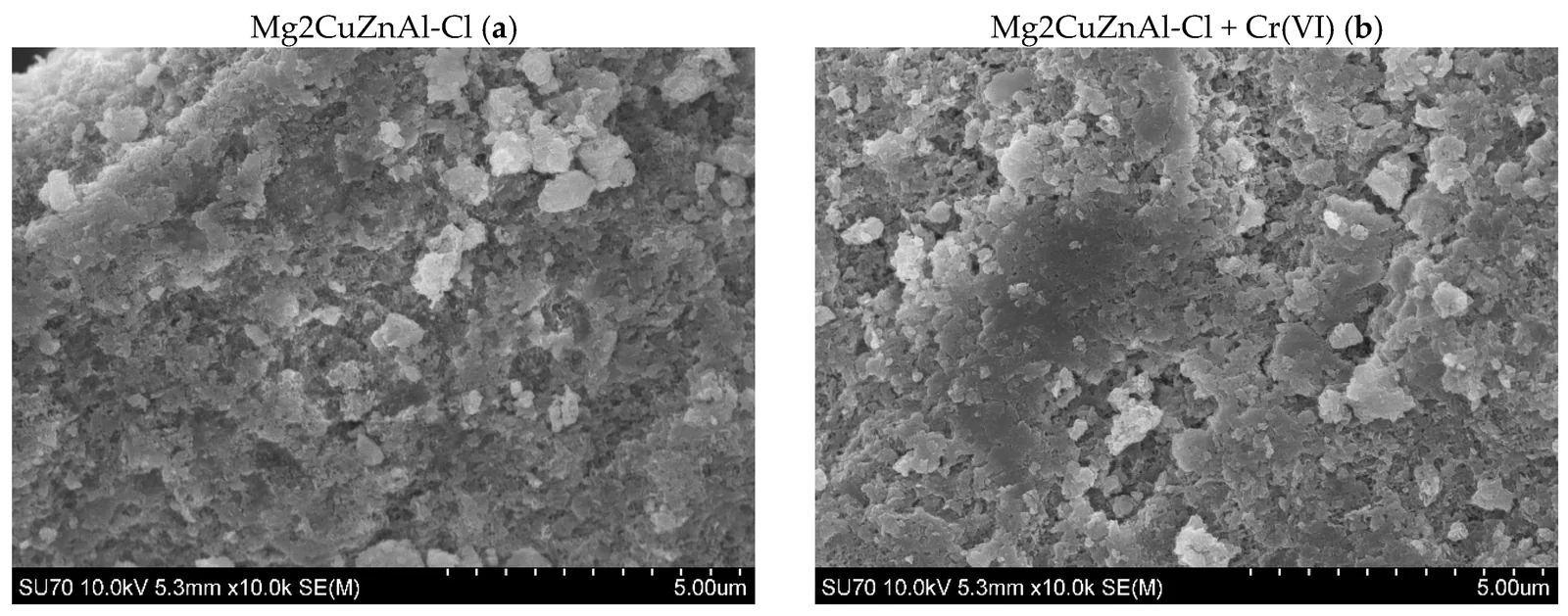
SEM images of Mg2CuZnAl–Cl sample before (**a**) and after (**b**) Cr(VI) adsorption.

SEM images (Figure 12 and Figure S2—Supplementary Information) show the morphology of the Mg2CuZnAl–Cl sample surface before and after Cr(VI) ion adsorption. In the case of the sample before adsorption, a plate-like structure typical of hydrotalcite materials is visible, consisting of irregular, thin plates forming loose agglomerates. Interlayer spaces are also visible, indicating a developed specific surface area and a preserved layered structure formed during the co-precipitation process. After adsorption of Cr(VI) ions, the surface of Mg2CuZnAl–Cl undergoes significant morphological changes. The plate-like structure becomes less pronounced, and the surface takes on a flatter appearance. Numerous heterogeneous smaller lumps and agglomerates are visible, forming a more compact layer covering the surface. This may be due to the fact that Cr-O-M (M = Mg, Cu, Zn, Al) complexes are formed on the outer surface in the form of a fine sediment. These deposits produce a smoothing effect in SEM [46]. However, the more compact and smoothed appearance refers to the local external morphology observed at the SEM scale and does not necessarily imply a decrease in the N_2_-accessible porosity of the whole sample. Such surface smoothing may coexist with changes in aggregate packing and increased accessibility of internal and interparticle mesoporous spaces [47]. Unlike SEM, BET analysis probes the surface accessible to N_2_ throughout the powder, including pore walls and interparticle voids. This provides a plausible explanation for the moderate increase in BET surface area and pore volume after Cr(VI) adsorption.

### 3.11. XPS Analysis—Surface Composition and Cr(VI) Sorption Mechanism

XPS analysis was performed on Mg2CuZnAl–Cl, Mg2CuZnAl–CO3 samples, and after Cr(VI) sorption, and the results are presented in Table 6. The survey spectra confirmed the presence of Mg, Cu, Zn, Al, O, and C in all tested materials. Chlorine was detected only in chloride form, while the chromium signal appeared only in samples after the sorption process. No additional signals indicating the presence of a separate NaCl crystalline phase were observed, suggesting that chlorine is mainly bound to the LDH structure and does not occur as a separate phase. The presence of carbon in all samples results both from the carbonate anions in the structure and from the adsorption of CO_2_ from the air and organic contaminants (C–C/C–H component). LDHs show a high affinity for atmospheric CO_2_, which is why partial carbonation of the surface is a typical phenomenon, also for the chloride form. Deconvolution of the O 1s spectra revealed the presence of O 1s A, B, C, H_2_O components, corresponding to: lattice oxygen in M–O–M bonds (M = Mg, Cu, Zn, Al) [48], metal hydroxyl groups, interlayer anions (CO_3_^2−^ and CrO_4_^2−^), and interlayer water [49,50,51,52]. After sorption of Cr(VI) in the form of Mg2CuZnAl-Cl and Mg2CuZnAl-CO3, a shift of the main O 1s maximum from approximately 531.6 eV to approximately 532.3 eV is observed. The shift towards higher binding energies indicates a change in the local electron environment of oxygen and the interaction of chromate anions with hydroxyl groups in brucite layers [10,46,53]. This interpretation is consistent with the FTIR results, which show characteristic Cr–O vibration bands, and with the XRD analysis, which confirms the preservation of the ordered layered structure without the formation of new crystalline phases containing chromium. This means that the changes observed in O 1s result from modifications in surface chemistry rather than from a phase transition of the material.

Two components are visible in the Cu 2p spectra (Figure S3—Supplementary Information): Cu2p_3/2_ (lower energy 930–935 eV) and Cu2p_1/2_ (higher energy 950–960 eV), resulting from spin–orbit coupling characteristic of Cu^2+^ ions [54,55,56,57,58,59]. Paramagnetic Cu^2+^ ions are characterized by the presence of satellite peaks at higher energies (usually 8–10 eV above the main peak). In the case under consideration, these are peaks around 940–945 eV. Multiplet splitting arises as a result of exchange interactions between unpaired valence electrons in the d shell of a transition metal (such as Cu) and core electrons during photoionisation, leading to the appearance of multiplet peaks (sat1, 2, 3 peaks). Multiplet splitting is crucial for distinguishing between the oxidation states of Cu(I) (d^10^ configuration) and Cu(II) (d^9^ configuration) and, in the case under consideration, indicates the absence of Cu(I) ions in the samples, which indicates that there was no reduction of copper during the sorption process. In the case of the Mg2CuZnAl–Cl sample after Cr(VI) sorption, clear shifts in the energy of the Cu 2p_3/2_ and Zn 2p_3/2_ peaks (of the order of 1–1.5 eV) are observed, which indicates a change in the coordination environment of Cu^2+^ and Zn^2+^ ions. In the carbonate form, these shifts are minimal, indicating less involvement of these metal centres in the chromate binding process. The shift of the Cu 2p_3/2_ peak towards higher energies following Cr(VI) adsorption indicates a reduction in the electron density on the Cu atoms and, consequently, an increase in the energy of the 2p orbitals; this in turn leads to the conclusion that there is no exchange of hydroxyl groups in ≡Cu-OH with HCrO_4_^−^, but rather the formation of an outersphere complex as follows: ≡Cu–OH ··· O=CrO_3_^2−^. The bond that forms polarises the O–H bond by drawing electron density away from the oxygen atoms, and indirectly also from Cu, towards the electronegative Cr, which consequently leads to an increase in the binding energy of the Cu 2p_3/2_ orbitals.

The position of the Al 2p peak remains virtually unchanged in the chloride form, which indicates that the integrity of the brucite layer has been preserved. Minor changes observed in the carbonate form may indicate a subtle reorganisation of the structural environment, but not degradation of the layer.

No significant changes in the relative intensity of Mg and Al signals were observed, suggesting no significant dissolution of magnesium during the anion exchange and sorption stages. This conclusion is consistent with the results of ICP analysis and the preservation of the layered structure confirmed by XRD and thermal analysis.

In the high-resolution Cr 2p spectra of Mg2CuZnAl–Cl + Cr(VI) and Mg2CuZnAl–CO_3_ + Cr(VI), the Cr 2p_3/2_ and Cr 2p_1/2_ components were observed at approximately 579.3–579.4 eV and 588.6–588.7 eV, respectively (Figure S4, Supplementary Information). These binding energies are consistent with Cr(VI) [60]. No distinct spectral components attributable to Cr(III) were detected. Thus, within the detection limits of XPS, no spectroscopic evidence of Cr(VI) reduction during adsorption was found. Atomic composition analysis using XPS enabled quantitative assessment of the changes occurring on the surface. For the Mg2CuZnAl–Cl sample before sorption, the Cl/Al ratio is approximately 0.32 (2.7/8.5). After Cr(VI) sorption, this value drops to approximately 0.07 (0.7/10.2), i.e., more than four times. At the same time, a Cr/Al ratio of approximately 0.05 (0.5/10.2) appears. The clear decrease in Cl/Al and the appearance of a positive Cr/Al ratio provide quantitative evidence of the partial replacement of Cl^−^ ions by chromate anions in the surface layer of LDH [40]. At the same time, the fact that chlorine does not disappear completely indicates that anion exchange is not a complete process and coexists with other binding mechanisms.

A comparison of XPS results with equilibrium data and XRD, FTIR, and BET data leads to the determination of a sorption mechanism that differs depending on the form of LDH. In the chloride form of Mg2CuZnAl, the Cr(VI) sorption process involves partial anion exchange (Cl^−^ ⇿ CrO_4_^2−^), confirmed by a change in the Cl/Al and Cr/Al ratios, and modification of the Cu^2+^ and Zn^2+^ coordination environment, indicating the participation of surface complexation while maintaining the integrity of the LDH layered structure. In contrast, in the carbonate form Mg2CuZnAl–CO3, strongly bound CO_3_^2−^ anions limit interlayer exchange, and the sorption process occurs mainly through surface interactions and coordination with hydroxyl groups. In both forms, there is also a physical sorption mechanism, i.e., electrostatic and van der Waals interactions, as indicated by the Dubinin-Radushkevich adsorption energy value (13–16.4 kJ/mol). The results obtained therefore confirm a mixed sorption mechanism involving both physical and chemical sorption, i.e., interlayer exchange and surface complexation, while maintaining the hydrotalcite structure of the material.

This mechanistic interpretation should also be considered in conjunction with the results of the leaching and sorption characterisation. Despite the observation that Mg, Cu, and Zn were being released, characteristic LDH reflections remained visible in the XRD patterns following Cr(VI) adsorption. This indicates that leaching did not lead to a complete collapse of the layered structure. Instead, the changes observed in the BET parameters and SEM images suggest local modifications to the pore structure, particle aggregation, and surface chemistry. Therefore, the release of structural cations is interpreted as a partial, pH-dependent destabilisation of the LDH, rather than a complete degradation of the material. Detailed adsorption and characterisation experiments were carried out at an initial pH of 7.2 and a Cr(VI) concentration of 0.5 mmol/L to ensure common reference conditions for comparing adsorption, leaching, and post-adsorption properties. A near-neutral pH was selected to limit the pronounced acid-induced dissolution observed at lower pH values, whilst avoiding strong competition from OH^−^ ions for adsorption sites under alkaline conditions. An initial Cr(VI) concentration of 0.5 mmol/L ensured measurable equilibrium concentrations and sufficient chromium uptake for a reliable posorption characterisation. These conditions were therefore used as model conditions enabling the interpretation of adsorption, speciation, leaching, and structural results within the same experimental system.

### 3.12. Comparison of the Investigated LDHs with Literature-Reported Cr(VI) Sorbents

In order to place the sorption properties of the tested Mg_2_Cu_0.5_Zn_0.5_Al_1_ materials in the context of previously described Cr(VI) sorbents, their adsorption capacities, experimental conditions, stability, desorption properties, and metal leaching were compared with data for selected LDH-based materials presented in the literature (Table 7). The comparison includes both unmodified LDHs and composites containing LDHs.

As shown in Table 7, the adsorption capacities of LDH-based materials used for Cr(VI) removal vary considerably due to differences in both material composition and experimental conditions. Therefore, a direct ranking of the sorbents based solely on q_max_ values is not fully justified. The adsorption capacity of 70 mg/g obtained for Mg_2_Cu_0.5_Zn_0.5_Al_1_–Cl indicates that this material exhibits competitive Cr(VI) adsorption performance, whereas the carbonate form showed a lower capacity of 34 mg/g. However, it should be emphasised that material stability and the leaching of structural cations were not systematically investigated in most of the studies included in the comparison. The present study therefore extends the conventional evaluation of sorbent performance by simultaneously considering Cr(VI) adsorption, the effect of the interlayer anion, pH-dependent leaching of structural cations, post-sorption material characterisation, and desorption behaviour. This approach enables LDH materials to be evaluated not only in terms of their maximum adsorption capacity, but also with regard to their stability and potential practical applicability.

## 4. Conclusions

Multication Mg–Cu–Zn–Al- and Mg-Al-Fe-layered double hydroxides (LDHs) were successfully synthesised by co-precipitation and evaluated as model systems for Cr(VI) adsorption. Among the investigated materials, Mg_2_Cu_0.5_Zn_0.5_Al_1_ exhibited consistently high Cr(VI) uptake under the screening conditions and was selected for further investigation of the influence of the interlayer anion. The results demonstrate that the type of interlayer anion plays a key role in controlling adsorption behaviour and structural stability of LDHs. The chloride form showed higher Cr(VI) uptake (70 mg/g), which can be attributed to easier anion exchange, whereas the carbonate form exhibited lower adsorption capacity (34 mg/g) but significantly greater structural stability due to stronger interlayer binding. Mg_2_Cu_0.5_Zn_0.5_Al_1_–Cl may be more suitable for applications where a high adsorption capacity for Cr(VI) is a priority, and where process conditions—in particular pH and metal leaching—can be controlled. Conversely, Mg_2_Cu_0.5_Zn_0.5_Al_1_–CO_3_ may be preferred in systems where the structural stability of the material and the limitation of metal cation release are more important than achieving maximum adsorption capacity. However, the practical application of both materials in multicomponent solutions and real water samples requires further verification. Adsorption proceeds via a mixed mechanism involving electrostatic interactions, anion exchange, and surface complexation, as supported by adsorption energy values (13–16.4 kJ/mol) and XPS analysis. The adsorption process is strongly influenced by pH, with higher efficiency observed under acidic conditions due to favourable chromate speciation and reduced competition with OH^−^ ions.

Differences between chloride and carbonate forms are also reflected in surface charge behaviour and electrokinetic stability. The carbonate form maintains a more stable surface charge over a wider pH range, whereas the chloride form exhibits higher interfacial reactivity but lower stability. These observations are consistent with leaching results, which confirm improved structural durability of the carbonate-intercalated LDH.

The results are consistent with the interaction of Cr(VI) species with surface functional groups and the possible involvement of Cu-containing sites, while no evidence of new crystalline chromium-containing phases was found.

The combined analysis of adsorption, surface, and stability properties reveals a clear correlation between adsorption capacity and structural stability, which is determined by the type of interlayer anions. This characteristic provides a more comprehensive basis for assessing LDH performance, in addition to adsorption capacity.

## Figures and Tables

**Table 1:** ICP-OES parameters.

Curve Type	Linear
Wavelength	Al-167.019 nm Cu-327.395 nm Mg-279.553 nm Zn-213.857 nm
Power	1 kV
Plasma flow	15 L/min
Auxiliary flow	1.5 L/min
Nebulizer flow	0.75 L/min
Replicate read time	1 s
Instrument stabilization delay	15 s
Sample uptake delay	16 s
Pump rate	15 rpm
Rinse time	10 s
Replicates	3

**Table 2:** Fitting parameters of experimental data using isotherm models: Freundlich, Langmiur, Langmuir-Freundlich, Dubinin-Radushkevich, Redlich Peterson, Temkin.

System	Isotherm Parameters	R^2^
Mg2CuZnAl-Cl	Redlich Peterson: A = 1.4 · 10^2^ (L/g), B = 1 · 10^5^ (L/mol), g = 0.84	0.9803
Mg2CuZnAl-CO3	Redlich Peterson: A = 48 (L/g), B = 2.6 · 10^4^ (L/mol), g = 0.72	0.9780
Mg2CuZnAl-Cl	Langmuir Freundlich: a_L-F_ = 1.35 · 10^−3^ (mol/g), K_L-F_ = 49 (L/mol), *n* = 0.42	0.9891
Mg2CuZnAl-CO3	Langmuir Freundlich: a_L-F_ = 6.45 · 10^−4^ (mol/g), K_L-F_ = 2.5 (L/mol), *n* = 0.34	0.9899
Mg2CuZnAl-Cl	Freundlich: K_F_ = 1.8 · 10^−3^ (L/g), *n* = 5.1	0.9694
Mg2CuZnAl-CO3	Freundlich: K_F_ = 1.9 · 10^−3^ (L/g), *n* = 3.4	0.9787
Mg2CuZnAl-Cl	Langmuir: a_L_ = 4.4 · 10^−4^ (mol/g), K_L_ = 7.6 · 10^4^ (L/mol)	0.7142
Mg2CuZnAl-CO3	Langmuir: a_L_ = 3.0 · 10^−4^ (mol/g), K_L_ = 6.6 · 10^3^ (L/mol)	0.8800
Mg2CuZnAl-Cl	Temkin: B = 4.33, b = 15.8, b_T_ = 0.57 (kJ/mol), K_T_ = 37.7 (L/mg)	0.9745
Mg2CuZnAl-CO3	Temkin: B = 3.74, b = 3.36, b_T_ = 0.66 (kJ/mol), K_T_ = 2.43 (L/mg)	0.9444
Mg2CuZnAl-Cl	Dubinin Raduschkevich: K_D-R_ = 1.87 · 10^−9^ (mol^2^/J^2^), lnQ_m_ = −7.12 (mol/g), E = 16.35 (kJ/mol)	0.9565
Mg2CuZnAl-CO3	Dubinin Raduschkevich: K_D-R_ = 2.76 · 10^−9^ (mol^2^/J^2^), lnQ_m_ = −7.45 (mol/g), E = 13.46 (kJ/mol)	0.9838

**Table 3:** Percentage desorption of chromate(VI) ions from carbonate and chloride forms of Mg2CuZnAl using selected desorbing agents (0.01 M Na_2_CO_3_, NaCl, NaOH, Na_2_HPO_4_) and distilled water, (pH_in_ and pH_eq_ refer to the pH of the solutions before and after desorption, respectively).

Desorbing Agent	pH_in_	Mg2CuZnAl–CO3	pH_eq_	Mg2CuZnAl–Cl	pH_eq_
Na_2_CO_3_	10.88	44.6%	10.78	78.2%	10.76
H_2_O	5.25	0.2%	8.16	4.8%	7.12
NaCl	6.64	3.6%	7.48	12.6%	7.52
NaOH	11.78	38.2%	11.75	56.2%	11.66
Na_2_HPO_4_	8.72	41.7%	8.68	56.8%	8.66

**Table 4:** Percentage adsorption (%A) and desorption (%D) of chromate(VI) ions for the carbonate and chloride forms of Mg2CuZnAl during three consecutive adsorption–desorption cycles. Adsorption was carried out from a 0.5 mmol/L Cr(VI) solution, while desorption was performed using a 10 mmol/L NaOH solution. The phase contact time for each step was 1 h.

Sorbent	Cycle 1		Cycle 2		Cycle 3	
	%A	%D	%A	%D	%A	%D
Mg2CuZnAl-CO3	36	39	25	27	25	14
Mg2CuZnAl-Cl	77	58	31	29	24	13

**Table 5:** Results of the BET analysis.

Sample	BET Surface Area (m^2^/g)	Pore Volume (cm^3^/g)	Average Pore Diameter (nm)
Mg2CuZnAl-CO3	56.65	0.262	18.65
Mg2CuZnAl-CO3 + Cr(VI)	47.85	0.262	21.90
Mg2CuZnAl-Cl	27.24	0.140	20.62
Mg2CuZnAl-Cl + Cr(VI)	30.69	0.172	22.43

**Table 6:** Surface composition (at.%) of Mg2CuZnAl samples determined by XPS based on survey spectra.

Sample	Element (Core Level)	Position	FWHM	%At Conc.	% St.Dev.
Mg2CuZnAl-Cl	C 1s	284.8	3.3	33.0	1.3
	O 1s	531.6	2.7	39.0	1.3
	O 1s A	531.0	1.5	11.3	1.3
	O 1s B	532.0	1.5	57.9	0.9
	O 1s C	533.0	1.5	27.4	0.4
	H_2_O	534.5	1.9	3.5	0.5
	Na 1s	1070.8	3.3	1.3	0.73
	Mg 2p	49.3	2.5	13.2	1.4
	Al 2p	74.1	3.7	8.5	0.63
	Cl 2p	198.6	3.3	2.7	0.71
	Cu 2p_3/2_	932.8	3.9	0.9	0.23
	Zn 2p_3/2_	1021.3	2.7	1.3	0.18
Mg2CuZnAl-CO3	C 1s	284.8	3.4	26.9	1.0
	O 1s	531.6	2.8	42.8	1.3
	O 1s A	531.0	1.5	20.5	0.5
	O 1s B	531.9	1.5	60.1	0.1
	O 1s C	533.0	1.5	16.9	0.9
	H_2_O	534.4	1.7	2.5	0.5
	Na 1s	1071.6	3.3	0.5	0.28
	Mg 2p	49.3	2.4	17.6	1.8
	Al 2p	74.1	4.5	10.1	0.56
	Cu 2p_3/2_	933.6	3.8	1.1	0.19
	Zn 2p_3/2_	1022.1	3.7	1.2	0.10
Mg2CuZnAl-Cl + Cr(VI)	C 1s	284.8	3.1	30.3	1.3
	O 1s	532.3	2.6	42.7	1.3
	O 1s A	530.9	1.5	16.9	0.9
	O 1s B	531.9	1.5	65.7	0.7
	O 1s C	533.0	1.5	15.9	0.9
	H_2_O	534.4	1.5	1.6	1.6
	Mg 2p	50.1	2.4	13.0	1.6
	Al 2p	74.1	4.0	10.2	0.81
	Cl 2p	198.6	3.0	0.7	0.72
	Cr 2p	577.3	5.4	0.5	0.14
	Cr 2p_3/2_	579.3	1.8	50.9	-
	Cr 2p_1/2_	588.6	1.8	49.1	-
	Cu 2p_3/2_	934.3	3.8	1.1	0.21
	Zn 2p_3/2_	1022.8	3.3	1.4	0.16
Mg2CuZnAl-CO3 + Cr(VI)	C 1s	284.8	3.2	27.1	1.1
	O 1s	532.3	2.6	44.9	1.4
	O 1s A	530.9	1.5	17.7	17.7
	O 1s B	531.9	1.5	64.9	64.9
	O 1s C	532.9	1.5	15.6	15.6
	H_2_O	534.4	1.3	1.8	1.8
	Mg 2p	50.1	2.4	15.5	1.6
	Al 2p	74.8	3.2	9.6	0.63
	Cr 2p	572.8	12.1	0.4	0.11
	Cr 2p_3/2_	579.4	1.8	59.2	-
	Cr 2p_1/2_	588.7	1.8	57.2	-
	Cu 2p_3/2_	933.6	3.1	1.2	0.24
	Zn 2p_3/2_	1022.8	2.8	1.3	0.19

**Table 7:** Comparison of the Cr(VI) removal performance of the investigated Mg_2_Cu_0.5_Zn_0.5_Al_1_ LDHs with selected LDH-based sorbents reported in the literature.

Sorbent	Selected Experimental Conditions	Max Adsorption Capacity, q_max_ [mg/g]	Stability/Reusability	Metal Leaching	Ref.
Mg–Al–CO_3_ hydrotalcite	Uncalcined LDH; batch sorption–desorption experiments	17	Sorption–desorption investigated	Not reported	[61]
Chromotropic-acid-intercalated Zn–Al LDH	*c_in_* = 200–10,000 mg/L	782	Not reported	Not reported	[62]
Ni_4_Fe_1_-LDH	*c_in_* = 4 mg/L; ca. 97% removal within 1 h	26.78	Not reported	Not reported	[63]
Exfoliated LDH nanosheets	308 K; equilibrium reached within approximately 8 min; pH 6	125.97	Not reported	Not reported	[64]
Calcined Ni/Mg/Al-LDH	Calcined hierarchical LDH-derived oxide; 30 °C	103.4	Not reported	Not reported	[65]
GO–NiFe-LDH composite	*c_in_* = 20 mg/L; 303 K; contact time 280 min	53.6	Not reported	Not reported	[66]
Ni/Fe-LDH; Glicerol-modified Ni/Fe LDH/GL	pH 5; equilibrium in approximately 30 min	50.43; 136.05	Structural stability after adsorption examined; Regeneration tested for seven cycles; improved chemical stability	Not reported	[67]
Calcined Mg/Al-LDH–polyaniline composite	pH 2	409.77	Not reported	Not reported	[68]
Ca–Al LDH	318 K	34.06	Not reported	Not reported	[69]
Polypyrrole-modified Ca–Al LDH	318 K; equilibrium in approximately 180 min	66.14	Not reported	Not reported	[69]
Polydopamine-modified MgAl-LDH	pH 2; PDA/LDH mass ratio 1:10	87	Reusability investigated	Not reported	[70]
Ultrasonically prepared Fe/Al-LDH	pH range 2–10; *c_in_* = 100 mg/L; dose 1 g/L	118.65	Stability of Cr-loaded material examined	Total Cr release from the spent material: 0.1052 mg/L after 30 days; structural-cation leaching	[71]
MgAl-LDH-modified plant-derived biochar	Equilibrium in approximately 180 min	177.88	Desorption approximately 85% after five cycles; resistance to competing ions investigated	Not reported	[72]
Fe_3_O_4_@MgAl-LDH	Magnetic composite; saturation magnetisation 34.53 emu/g	108.7	Magnetic separation and reuse demonstrated	Not reported	[73]
Porous NiCo-LDH microspheres	Room temperature	123	Cr(VI) removal remained approximately 82% after five cycles	Not reported	[45]
ZnAl-LDH/SiO_2_ composite	pH 5.5; 298 K; sorbent dose 1 g/L for isotherm experiments	176	Cr(VI) removal decreased from 98.9% to 92.5% after six cycles	Not reported	[74]
Mg_2_Cu_0.5_Zn_0.5_Al_1_–Cl,	*c_in_* = 0.5 mmol/L; pH = 7.2; room temperature; contact time t = 1 h	70	Up to 78.2% desorption with 0.01 M Na_2_CO_3_	Lower stability; Higher Mg, Cu, and Zn release, particularly under acidic conditions	this study
Mg_2_Cu_0.5_Zn_0.5_Al_1_–CO_3_	*c_in_* = 0.5 mmol/L; pH = 7.2; room temperature; contact time t = 1 h	34	Up to 44.6% desorption with 0.01 M Na_2_CO_3_	Greater stability and Lower release of structural cations	this study

## Data Availability

The original contributions presented in this study are included in the article/Supplementary Material. Further inquiries can be directed to the corresponding author.

## Supplementary Materials

The following supporting information can be downloaded at: https://www.mdpi.com/article/10.3390/ma19163366/s1, Figure S1: Sorption isotherms for Cr(VI) ions on Mg2CuZnAl-CO3 (a) and Mg2CuZnAl-Cl (b) forms of sorbent; Figure S2: SEM images of Mg2CuZnAl–Cl sample obtained at different magnifications before (a) and after (b) Cr(VI) adsorption; Figure S3: Representative XPS Cu2p spectra fitted with contributions from CuO/CuCl_2_ and CuCO_3_; Cu 2p_3/2_ (930–935 eV) and Cu 2p_1/2_ (950–960 eV) spin-orbit components and Cu^2+^ satellite features at ~940–945 eV are indicated; Figure S4: XPS Cr2p region, overlaping with the broad Cu LMM Auger features; the Cr2p_3/2_ and Cr2p_1/2_ peak positions indicates exclusively Cr(VI) in the samples; Table S1: XRD results.

## Abbreviations

The following abbreviations are used in this manuscript:

BET	Brunauer–Emmet–Teller method
c_eq_	concentration of Cr(VI) ions in the solutions after sorption (mol/L)
c_in_	concentration of Cr(VI) ions in the initial solution (mol/L)
FTIR	Fourier transform infrared spectroscopy
ICP-OES	inductively coupled plasma optical emission spectrometry
LDH	layered double hydroxide
LDH-Cl	simplified notation for chloride form of layered double hydroxide used in this work
LDH-CO3	simplified notation for carbonate form of layered double hydroxide used in this work
Mg2CuZnAl	simplified notation for Mg_2_Cu_0.5_Zn_0.5_Al_1_ layered double hydroxide used in this work
Mg2CuZnAl-Cl	simplified notation for Mg_2_Cu_0.5_Zn_0.5_Al_1_ layered double hydroxide in chloride form used in this work
Mg2CuZnAl-CO3	simplified notation for Mg_2_Cu_0.5_Zn_0.5_Al_1_ layered double hydroxide in carbonate form used in this work
pH_eq_	pH of the solution at equilibrium
pH_in_	initial pH of the solution
pzc	zero charge point
SEM	scanning electron microscopy
XPS	X-ray photoelectron spectroscopy
XRD	X-ray diffraction analysis
