# Supplementary material for: MgCuZnAl-Layered Double Hydroxides for Cr(VI) Adsorption: Effect of Interlayer Anions on Uptake Behavior and pH-Dependent Stability
Source: Materials (Basel). 2026 Aug 7;19(16):3366. doi: 10.3390/ma19163366 (PMC13514817; doi:10.3390/ma19163366)
Supplement: Supplementary file 1 [file materials-19-03366-s001.zip › Figure_S1.pdf]

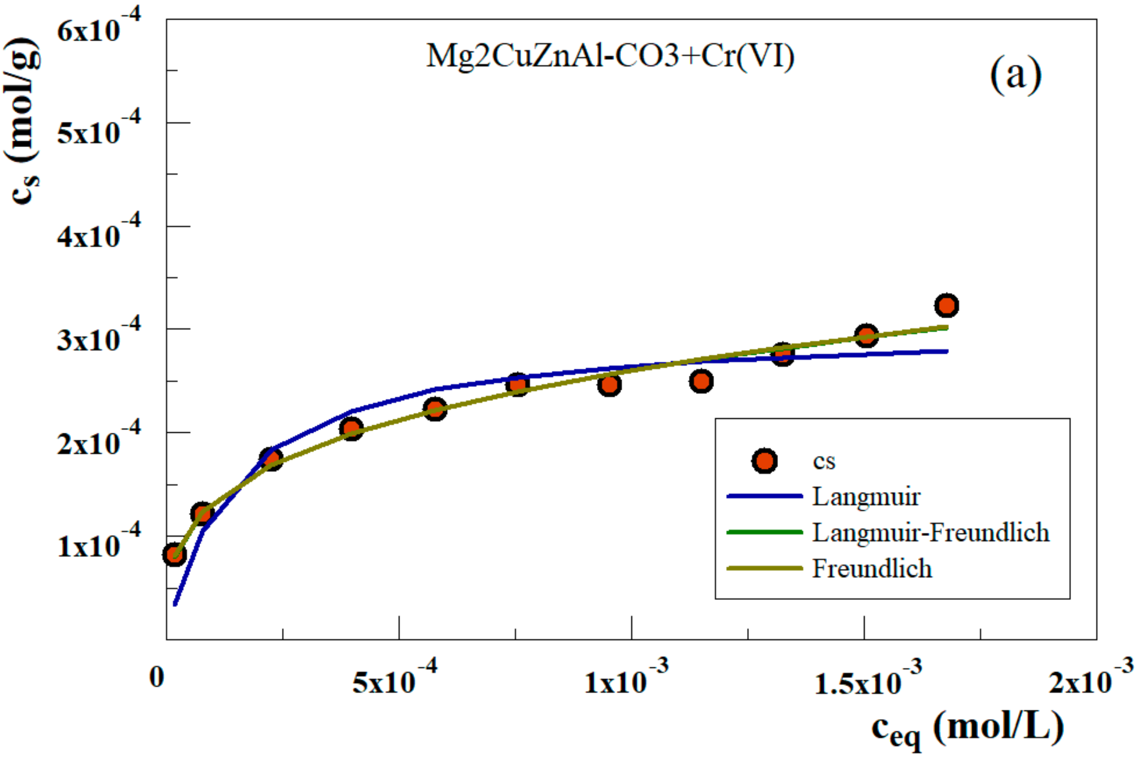

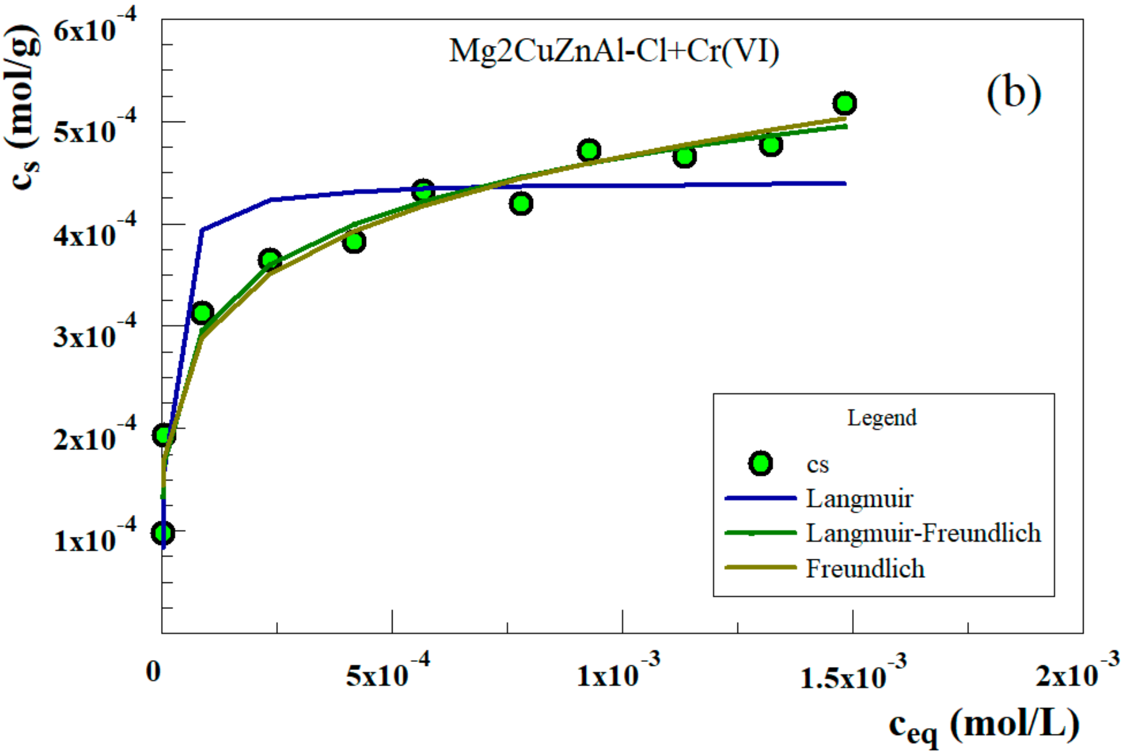

Figure S1. Sorption isotherms for Cr(VI) ions on Mg2CuZnAl-CO3 (a) and Mg2CuZnAl-Cl (b) forms of sorbent.
