# Supplementary material for: MgCuZnAl-Layered Double Hydroxides for Cr(VI) Adsorption: Effect of Interlayer Anions on Uptake Behavior and pH-Dependent Stability
Source: Materials (Basel). 2026 Aug 7;19(16):3366. doi: 10.3390/ma19163366 (PMC13514817; doi:10.3390/ma19163366)
Supplement: Supplementary file 1 [file materials-19-03366-s001.zip › Figure_S2.pdf]

Mg2CuZnAl-Cl (a)	Mg2CuZnAl-Cl+Cr(VI) (b)

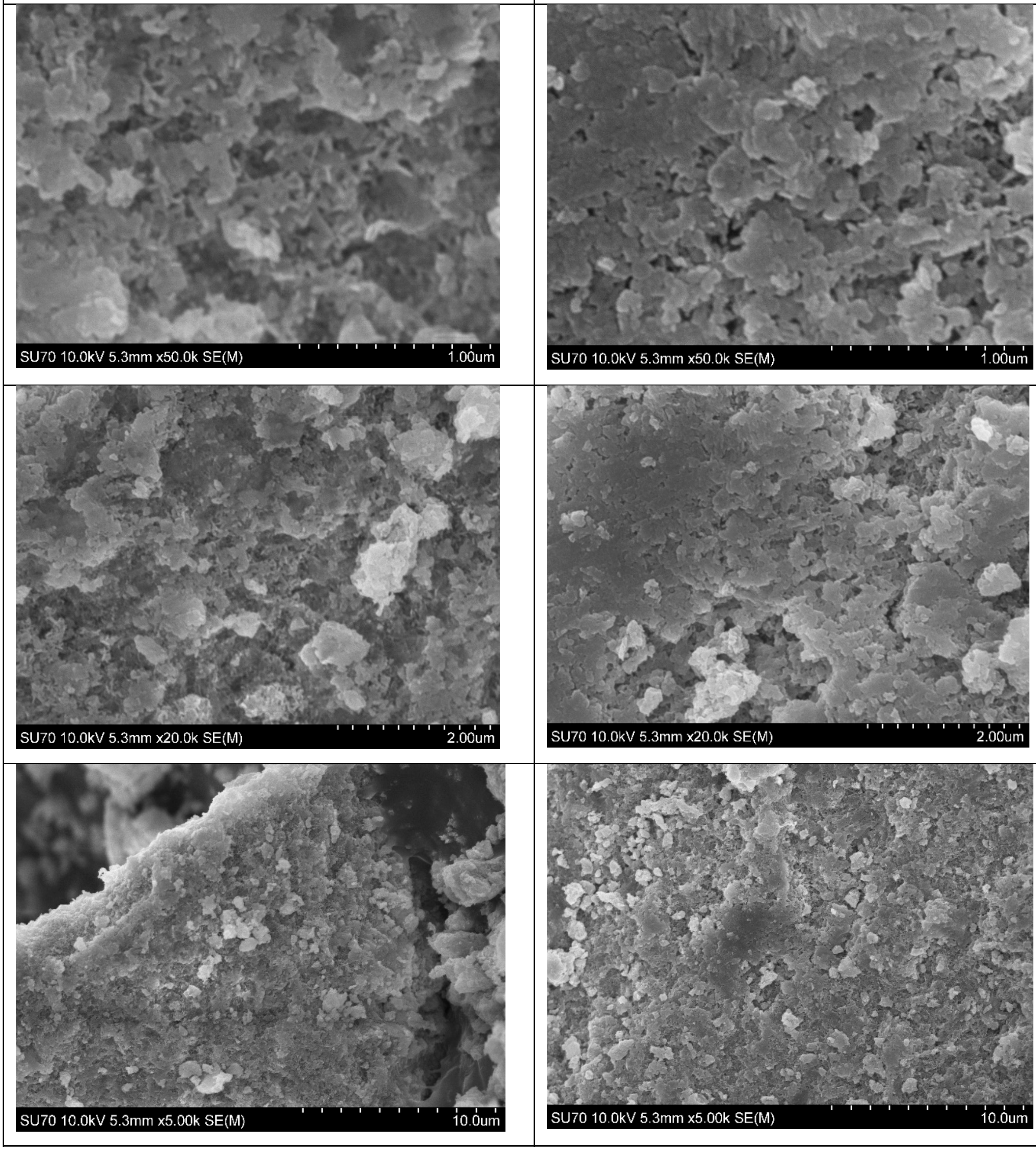

Figure S2. SEM images of Mg2CuZnAl–Cl sample obtained at different magnifications before (a) and after (b) Cr(VI) adsorption.
