# Supplementary material for: MgCuZnAl-Layered Double Hydroxides for Cr(VI) Adsorption: Effect of Interlayer Anions on Uptake Behavior and pH-Dependent Stability
Source: Materials (Basel). 2026 Aug 7;19(16):3366. doi: 10.3390/ma19163366 (PMC13514817; doi:10.3390/ma19163366)
Supplement: Supplementary file 1 [file materials-19-03366-s001.zip › Figure_S3.pdf]

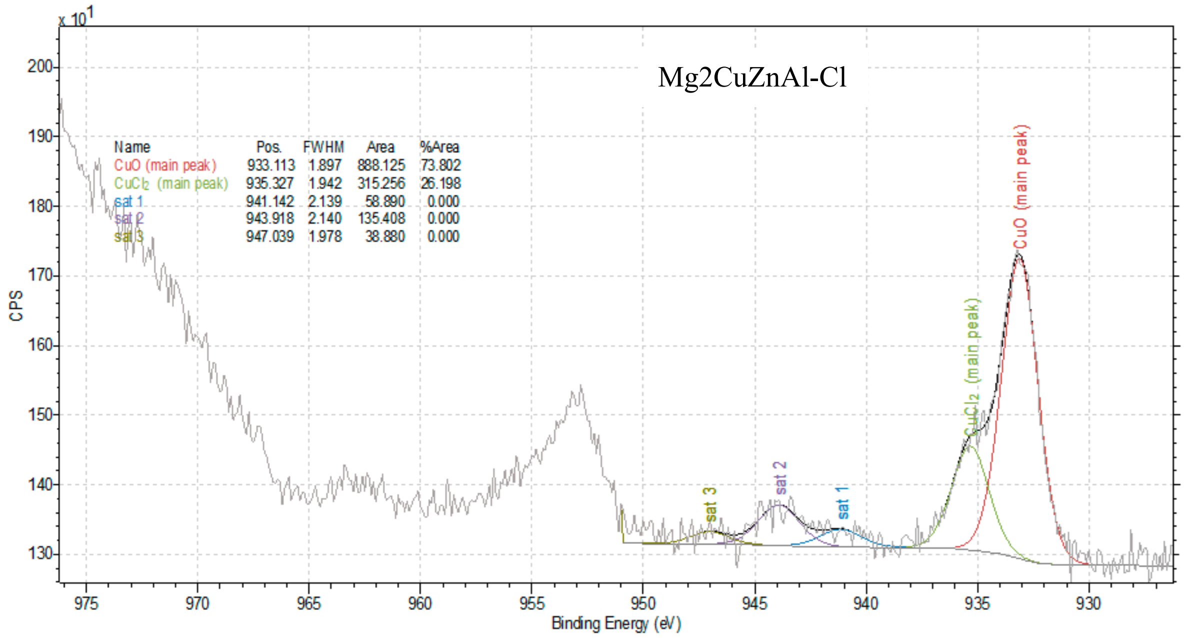
Mg2CuZnAl-Cl
x 10^1
CPS
Binding Energy (eV)
Name | Pos. | FWHM | Area | %Area
CuO (main peak) | 933.113 | 1.897 | 888.125 | 73.802
CuCl2 (main peak) | 935.327 | 1.942 | 315.256 | 26.198
sat 1 | 941.142 | 2.139 | 58.890 | 0.000
sat 2 | 943.918 | 2.140 | 135.408 | 0.000
sat 3 | 947.039 | 1.978 | 38.880 | 0.000
CuO (main peak)
CuCl2 (main peak)
sat 1
sat 2
sat 3

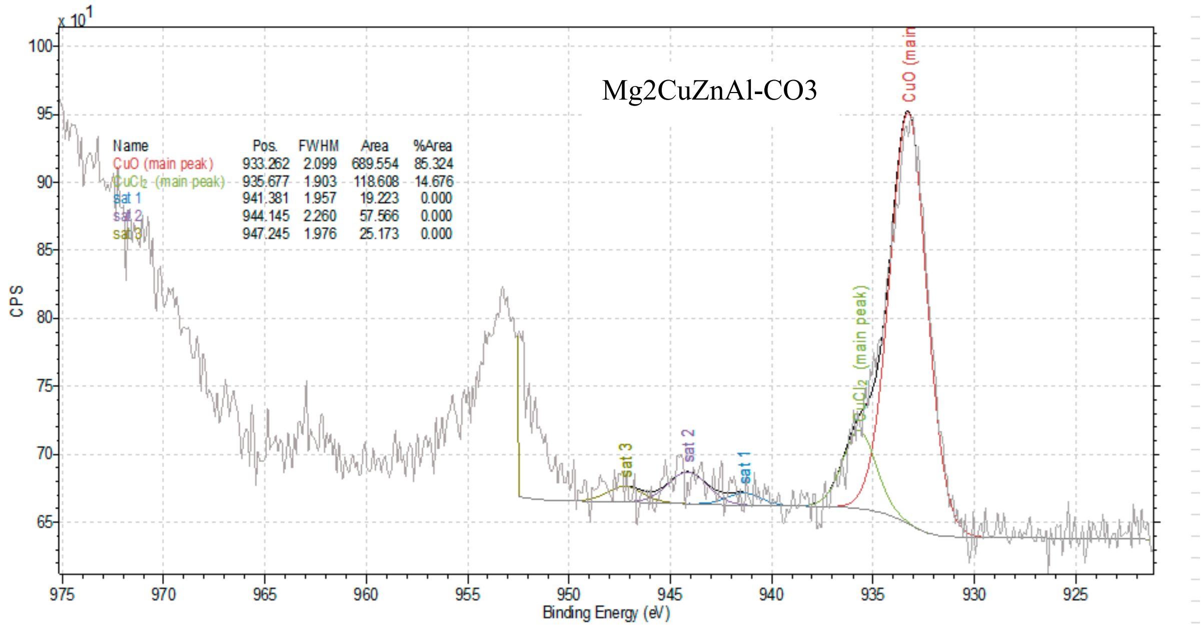
Mg2CuZnAl-CO3
x 10^1
CPS
Binding Energy (eV)
Name | Pos. | FWHM | Area | %Area
CuO (main peak) | 933.262 | 2.099 | 689.554 | 85.324
CuCl2 (main peak) | 935.677 | 1.903 | 118.608 | 14.676
sat 1 | 941.381 | 1.957 | 19.223 | 0.000
sat 2 | 944.145 | 2.260 | 57.566 | 0.000
sat 3 | 947.245 | 1.976 | 25.173 | 0.000
CuO (main
CuCl2 (main peak)
sat 1
sat 2
sat 3

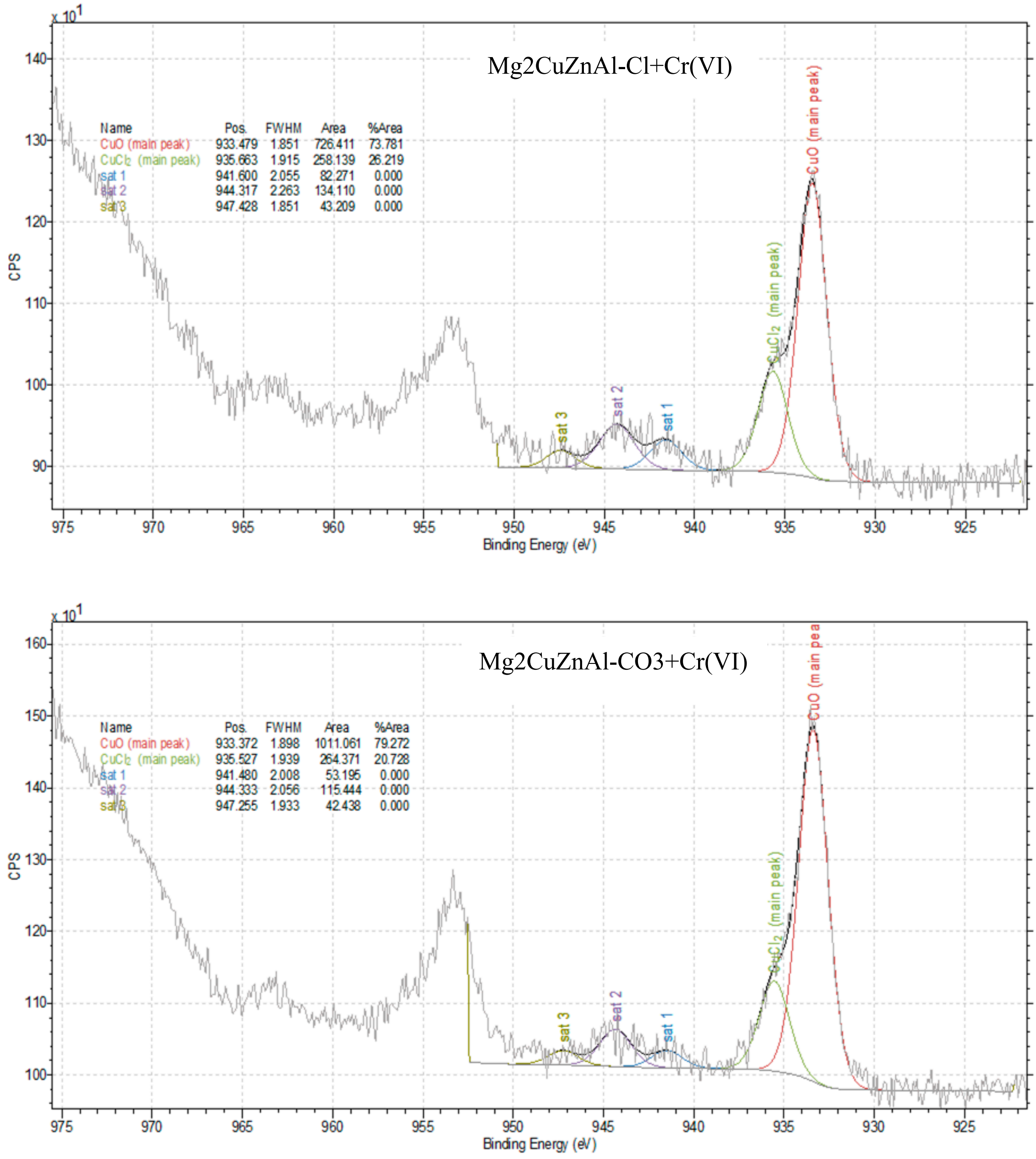

Figure S3. Representative XPS Cu2p spectra fitted with contributions from $CuO/CuCl_2$ and $CuCO_3$; Cu $2p_{3/2}$ (930-935 eV) and Cu $2p_{1/2}$ (950-960 eV) spin-orbit components and Cu^{2+} satellite features at ~940-945 eV are indicated.
