# Supplementary material for: MgCuZnAl-Layered Double Hydroxides for Cr(VI) Adsorption: Effect of Interlayer Anions on Uptake Behavior and pH-Dependent Stability
Source: Materials (Basel). 2026 Aug 7;19(16):3366. doi: 10.3390/ma19163366 (PMC13514817; doi:10.3390/ma19163366)
Supplement: Supplementary file 1 [file materials-19-03366-s001.zip › Figure_S4.pdf]

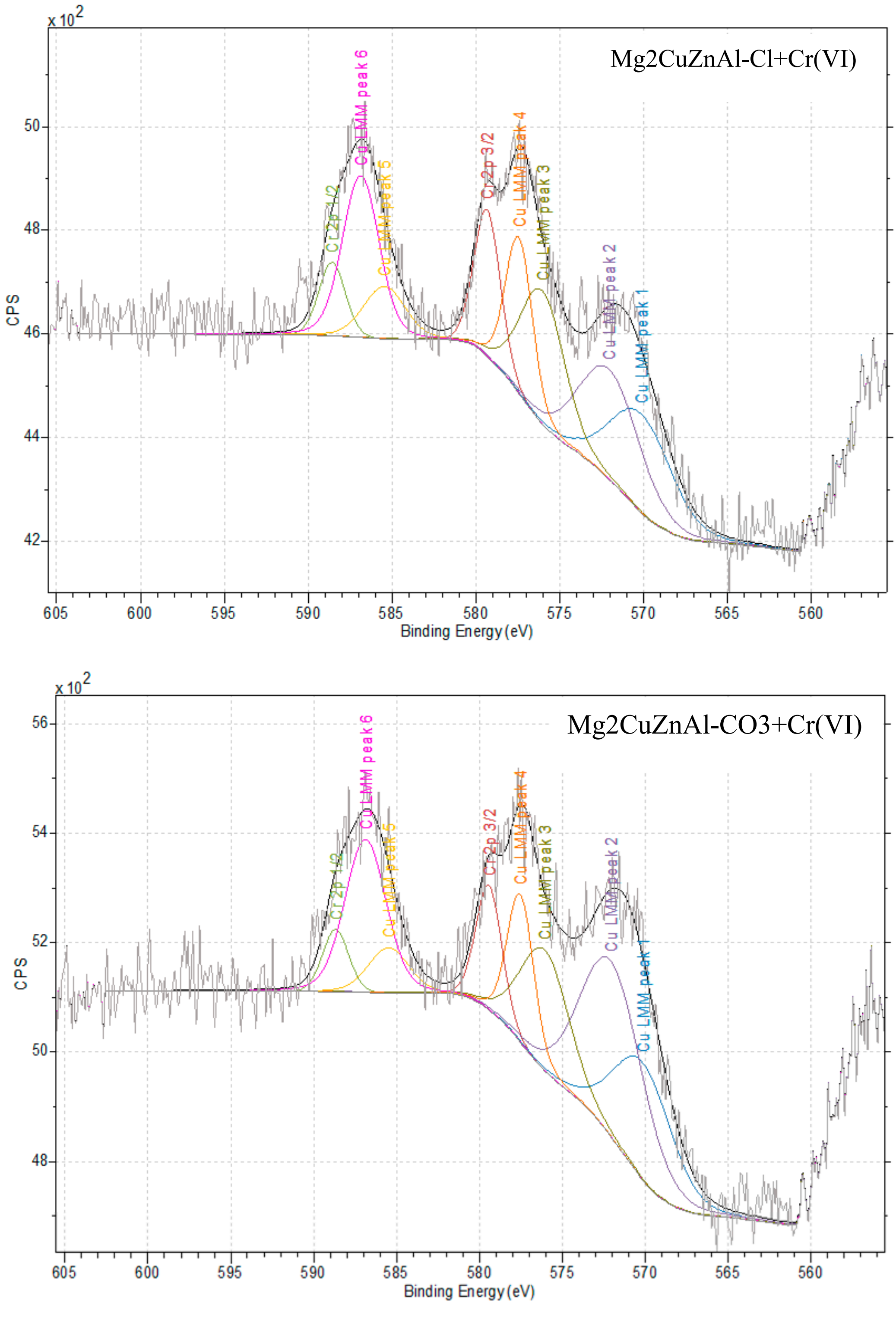

Figure S4. XPS Cr2p region, overlaping with the broad Cu LMM Auger features; the $Cr2p_{3/2}$ and $Cr2p_{1/2}$ peak positions indicates exclusively Cr(VI) in the samples.
