# Supplementary material for: MgCuZnAl-Layered Double Hydroxides for Cr(VI) Adsorption: Effect of Interlayer Anions on Uptake Behavior and pH-Dependent Stability
Source: Materials (Basel). 2026 Aug 7;19(16):3366. doi: 10.3390/ma19163366 (PMC13514817; doi:10.3390/ma19163366)
Supplement: Supplementary file 1 [file materials-19-03366-s001.zip › Table_S1.pdf]

Table S1. XRD results.

Sample	(003) 2θ	d(003) [Å]	FWHM (003)	(006) 2θ	FWHM (006)	(009) 2θ	(110) 2θ	(113) 2θ
Mg2CuZnAl–CO3	11.50	7.695	1.09	23.09	1.19	34.72	60.52	61.74
Mg2CuZnAl–CO3+Cr	11.35	7.796	1.06	22.97	1.36	34.58	60.40	61.61
Mg2CuZnAl–Cl	11.32	7.817	1.02	22.85	1.32	34.62	60.43	61.67
Mg2CuZnAl–Cl+Cr	11.11	7.964	1.17	22.75	1.42	34.36	60.24	61.42

Sample	d(110) [Å]	a = 2·d(110) [Å]	c = 3·d(003) [Å]
$Mg_2CuZnAl$–CO_3	1.530	3.060	23.085
$Mg_2CuZnAl$–CO_3+Cr	1.533	3.066	23.388
$Mg_2CuZnAl$–Cl	1.532	3.064	23.451
$Mg_2CuZnAl$–Cl+Cr	1.536	3.072	23.892
